# A Feedback Loop between Dynamin and Actin Recruitment during Clathrin-Mediated Endocytosis

**DOI:** 10.1371/journal.pbio.1001302

**Published:** 2012-04-10

**Authors:** Marcus J. Taylor, Marko Lampe, Christien J. Merrifield

**Affiliations:** 1 MRC Laboratory of Molecular Biology, Cambridge, United Kingdom; 2 CNRS UPR3082, Laboratoire d'Enzymologie et de Biochimie Structurales, Gif-sur-Yvette Cedex, France; The Scripps Research Institute, United States of America

## Abstract

Clathrin-mediated endocytosis proceeds by a sequential series of reactions catalyzed by discrete sets of protein machinery. The final reaction in clathrin-mediated endocytosis is membrane scission, which is mediated by the large guanosine triophosphate hydrolase (GTPase) dynamin and which may involve the actin-dependent recruitment of N-terminal containing BIN/Amphiphysin/RVS domain containing (N-BAR) proteins. Optical microscopy has revealed a detailed picture of when and where particular protein types are recruited in the ∼20–30 s preceding scission. Nevertheless, the regulatory mechanisms and functions that underpin protein recruitment are not well understood. Here we used an optical assay to investigate the coordination and interdependencies between the recruitment of dynamin, the actin cytoskeleton, and N-BAR proteins to individual clathrin-mediated endocytic scission events. These measurements revealed that a feedback loop exists between dynamin and actin at sites of membrane scission. The kinetics of dynamin, actin, and N-BAR protein recruitment were modulated by dynamin GTPase activity. Conversely, acute ablation of actin dynamics using latrunculin-B led to a ∼50% decrease in the incidence of scission, an ∼50% decrease in the amplitude of dynamin recruitment, and abolished actin and N-BAR recruitment to scission events. Collectively these data suggest that dynamin, actin, and N-BAR proteins work cooperatively to efficiently catalyze membrane scission. Dynamin controls its own recruitment to scission events by modulating the kinetics of actin and N-BAR recruitment to sites of scission. Conversely actin serves as a dynamic scaffold that concentrates dynamin and N-BAR proteins at sites of scission.

## Abstract 2

A live-cell imaging study reveals that a positive feedback loop between dynamin and actin contributes to efficient endocytic membrane scission.

## Author Summary

Cells internalize surface receptors via clathrin-mediated endocytosis, a process in which receptors concentrate in clathrin-coated pits in the plasma membrane that pinch into the cell as membrane vesicles. The mechanism by which vesicles pinch off from the plasma membrane is referred to as membrane scission and this requires the large guanosine triphosphate hydrolase (GTPase) dynamin, curvature sensing/inducing N-terminal helix containing Bin/Amphiphysin/Rvs (N-BAR) domain proteins and regulation by the actin cytoskeleton. The precise mechanism of dynamin recruitment to scission sites, the kinetics of this recruitment, and how dynamin regulates recruitment of its binding partners and scission is not well understood. In this study we used an optical assay to measure the recruitment kinetics of dynamin, actin, and N-BAR proteins BIN1 and endophilin2 to individual clathrin-mediated endocytic scission events. We show that there is a positive feedback loop between dynamin and actin at membrane scission sites: dynamin regulates actin recruitment and actin assembly stabilizes dynamin recruitment. The kinetics of dynamin, actin, and N-BAR protein recruitment are in turn regulated by dynamin's GTPase activity. Conversely, inhibiting actin dynamics decreases the incidence of scission and decreases the amplitude of dynamin recruitment to scission events by around 50%. Our data suggest that dynamin, actin, and N-BAR proteins cooperate to catalyze efficient membrane scission and that dynamin controls its own recruitment to scission by modulating the actin cytoskeleton.

## Introduction

Clathrin-mediated endocytosis (CME) begins when a clathrin-coated bud forms at the plasma membrane through the processive recruitment of cargo, adaptors, and accessory proteins including curvature inducing/sensing BIN/Amphiphysin/RVS domain (BAR) domain proteins [1]–[3]. The actin cytoskeleton may also play a role in the late stages of clathrin-coated bud formation in some cell types [4]–[7] through force generation and/or through segregation of lipids [8]–[10] (though see [11]). The process is completed when the constricted membrane neck of the clathrin-coated bud is severed to release a clathrin-coated vesicle (CCV) in a reaction involving guanosine triophosphate (GTP) hydrolysis by the large GTP hydrolase (GTPase) dynamin [12],[13].

Despite this timeline of the molecular dynamics of CCV formation, the underlying mechanisms that govern how and when endocytic proteins arrive and depart from sites of CME are still poorly understood. Pharmacological [14] and live cell imaging studies [15] in conjunction with dynamin mutants [16]–[18] have shown that the GTPase activity of dynamin is not solely involved in the scission reaction but also is functionally relevant at earlier time points during clathrin-coated bud maturation (see also [19],[20] for recent reviews). This finding suggests that the GTPase activity of dynamin could have a role in regulating the recruitment kinetics of endocytic proteins such as N-terminal containing BIN/Amphiphysin/RVS domain (N-BAR) proteins, actin, and actin effectors in the seconds preceding scission. In support of this hypothesis, N-BAR proteins can directly bind to dynamin [21],[22] and also promote the generation of highly curved membrane templates to which dynamin preferentially binds [23],[24]. This suggests a cooperative relationship with dynamin during membrane scission [3],[25]. Moreover dynamin recruitment and actin polymerization occur over a similar time course in the seconds preceding scission [26]. Thus the dynamin GTPase cycle could potentially modulate endocytic actin and N-BAR protein dynamics. It has been proposed that dynamin is a negative regulator of endocytic actin dynamics [27]. There is also evidence that the GTPase activity of dynamin is required for actin polymerization in some cellular contexts [28],[29] and dynamin can bind filamentous actin (F-actin) and promote actin polymerization via F-actin uncapping [30]. However, despite the circumstantial evidence, it remains unclear whether the dynamin GTPase cycle regulates actin polymerization and the recruitment dynamics of dynamin and key dynamin binding partners to clathrin-coated buds in the ∼20 s before scission in vivo. Conversely actin could play a role in concentrating dynamin at sites of scission because dynamin binds directly to F-actin [30] and F-actin binding effectors are implicated in actin polymerization [31],[32].

Here we investigated potential dependencies between dynamin, actin, and N-BAR protein recruitment during CME by employing the “pulsed pH assay”: a total internal reflection fluorescence microscopy (TIR-FM)–based assay that detects the internalization of transferrin receptor at individual clathrin-coated structures (CCS) [26],[33]. By using scission events as a spatial and temporal reference the kinetics of protein recruitment to sites of scission can be measured with 2-s resolution [26],[33]. We found that dynamin and the actin cytoskeleton have complementary regulatory functions during mammalian CME: dynamin GTPase activity regulated the kinetics of dynamin, actin, and N-BAR recruitment and, in turn, a dynamic actin cytoskeleton was required to concentrate dynamin and N-BAR proteins at sites of scission. Our data indicate that a feedback mechanism exists between dynamin and actin in the moments leading up to membrane scission during CME.

## Results

### Dynamin Recruitment to Sites of Scission

**Figure 1:**
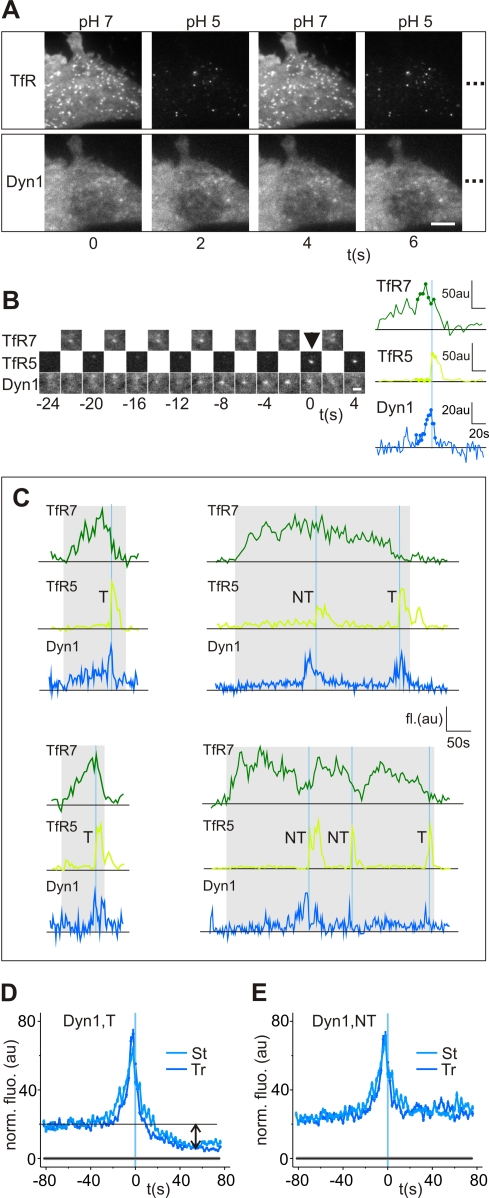
The kinetics of dynamin recruitment to sites of membrane scission. (A) Sequential images from a TIR-FM image series of an NIH-3T3 fibroblast stably expressing dyn1(WT)-mCherry (Dyn1, lower panel) and transiently expressing TfR-pHl (TfR, upper panel). CCS were marked by spots of TfR-phl (spots, TfR images, pH7). At pH5 the fluorescence of externally accessible TfR-SEphl at CCS and on the membrane quenched to reveal acid-resistant, internalized TfR-phl. (B) Dyn1(WT)-mCherry was recruited shortly before scission. Time series of an example CCS (marked by TfR7, upper panel), which hosted a scission event (indicated by arrow, TfR5 image series) to which dyn1(WT)-mCherry was recruited (lower image series). Quantified fluorescence measurements from the time series shown on right (dark green, TfR7; light green, TfR5; blue, Dyn1). Dots correspond to the images shown. Scission manifested as an abrupt increase in the TfR5 fluorescence signal as the endocytic vesicle was occluded from external acidification. (C) Example full-length TfR7 fluorescence traces of CCS showing associated scission events and dyn1(WT)-mCherry recruitment. Example events of CCS that hosted one scission event (two sets of traces on left), two scission events (set of traces upper right), or three scission events (set of traces lower right). Scission events were classified as “terminal” (T) or “non-terminal” (NT) depending on whether the host CCS persisted or disappeared following scission (see text for explanation). Grey boxes indicate time over which CCS were visible. (D–E) Ensemble recruitment signatures of dyn1(WT)-mCherry for terminal events (D) or non-terminal events (E). Similar recruitment kinetics were observed in cells expressing dyn1(WT)-mCherry either stably (light blue) or transiently (dark blue). Transient “flickering” of dyn1(WT)-mCherry manifested as elevated pre-scission dyn1(WT)-mCherry fluorescence (black line and arrow on (D)), which persisted in non-terminal events following scission (E). Error bars represent standard error of the mean. Grey lines in (D and E) represent 95% confidence limits for random fluorescence measurements (see Materials and Methods for details). (D, stable, 11 cells, 1,483 events; transient, 21 cells, 2,229 events; E stable, 11 cells, 1,483 events; transient, 21 cells, 2,229 events).

NIH-3T3 cells stably expressing WT mouse dynamin1 fused to mCherry (dyn1(WT)-mCherry) were transiently transfected with the reporter construct human transferrin receptor (TfR) fused to the pH-sensitive green fluorescent protein pHluorin (TfR-phl) [33]–[35]. Cells were assayed with the pulse pH assay by synchronizing image acquisition with local perfusion of a target cell with alternate low and high pH buffers (Figure 1A; in the following text TfR7 refers to the TfR-phl signal at pH7, and TfR5 refers to TfR-phl signal at pH5). The moment of vesicle formation was detected when a fluorescent puntcum of TfR-phl appeared in an image acquired at pH5 (TfR5 t = 0 s; Figure 1B; see Materials and Methods for description of quantification and [26] and [33] for details). A prominent burst of dyn1(WT)-mCherry recruitment was observed in the frames directly preceding the appearance of individual scission events (Figure 1B) [26]. Detailed signal analysis revealed the dyn1(WT) fluorescent signal consisted of low amplitude “flickering” that preceded the final burst of dynamin fluorescence that peaked 2–4 s before vesicle appearance (Figure 1C; also see Video S1).

Scission events were used as a fiducial marker to align dyn1(WT)-mCherry fluorescent recruitment signals and generate an average ensemble recruitment “signature”; a temporal readout of a protein's recruitment relative to scission. The recruitment signature reflects the dynamic equilibrium between protein free in the cytosol and accumulated at the CCS. The dyn1(WT)-mCherry ensemble recruitment signature peaked at 2 s before vesicle detection (t = 0; Figure 1D and 1E), consistent with previous measurements [26] and dynamin's role in catalyzing membrane scission [23],[36]. The average ensemble dyn1(WT)-mCherry recruitment signature was equivalent for NIH-3T3 cells that were transiently or stably expressing dyn1(WT)-mCherry (Figure 1D and 1E). For scission events defined as “terminal” (hosted by a parent CCS that disappeared after scission, see TfR7 channel in Figure 1C; see Materials and Methods; and also [26]), the low amplitude “flickering” observed at individual events manifested as an elevated pre-scission fluorescence, which disappeared following scission (see arrows in Figure 1D). In the recruitment signature to scission events defined as “non-terminal” (hosted by a parent CCS that persisted post-scission at the plasma membrane and could host multiple scission events; see TfR7 traces in Figure 1C) the elevated fluorescence persisted after scission, indicating continual dynamin flickering at the remaining portion of CCS (Figure 1E). The low amplitude “flickering” indicated that dynamin was present at CCS at early time points and suggested transient recruitment, with dynamin constantly “hopping” on and off CCS until time points close to scission when its recruitment was stabilized.

### Dynamin GTPase Mutants Have Different Effects on the Recruitment Kinetics of Dynamin to Scission

**Figure 2:**
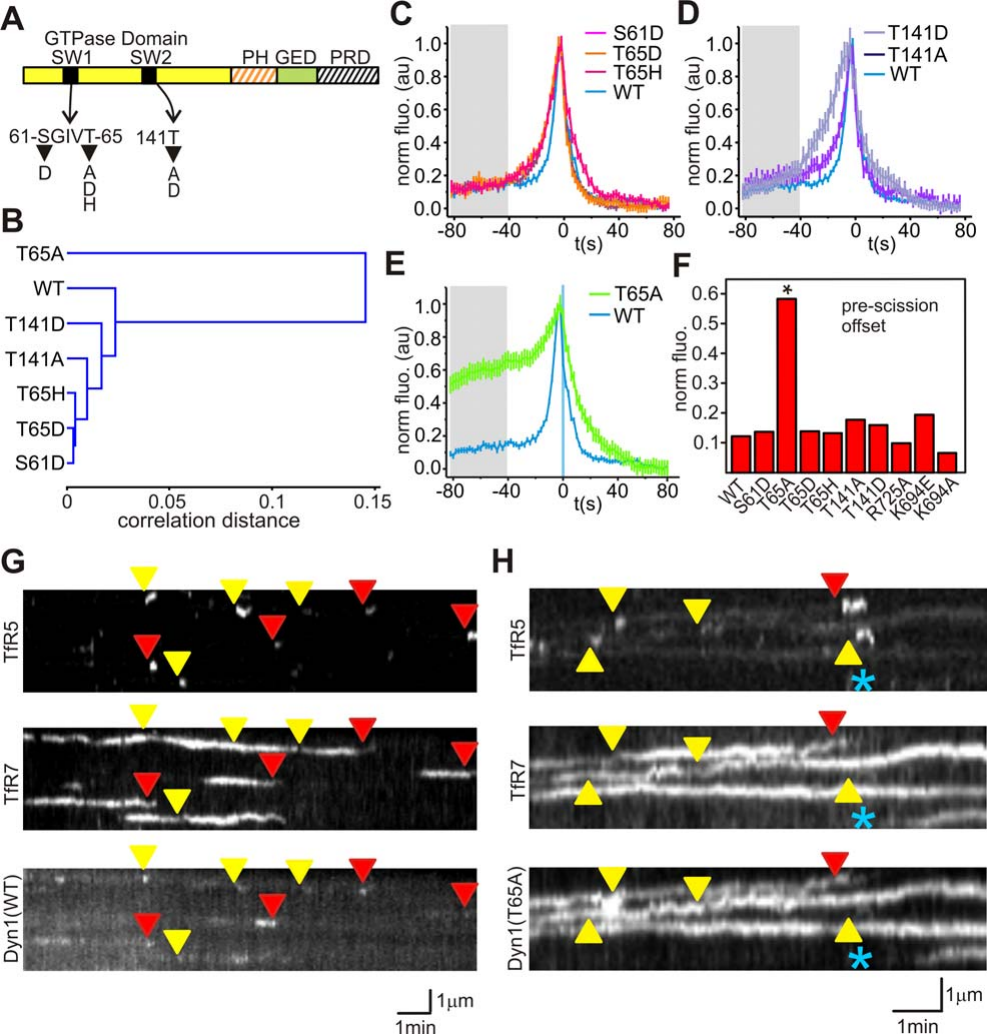
The effects of Dynamin GTPase mutants on Dynamin recruitment kinetics. (A) Domain architecture of dynamin showing the position of point mutation within the GTPase domain and the position of the pleckstrin homology domain (PH), guanine exchange domain (GED) and proline rich domain (PRD). The GTPase point mutations selected were within the switch1 and 2 (SW1 and SW2) regions of the GTPase domain and exhibited a range of Km and k_cat_ values (see text). (B–E) Dynamin GTPase point mutants with similar enzymatic constants showed similar recruitment kinetics to scission. The pre-scission recruitment profiles were compared pair-wise and arranged in a dendrogram by hierarchal clustering. (C–E) Comparison of dyn1(WT)-mCherry and GTPase mutants recruitment signatures. (C) Dyn1(T65D)-mCherry, dyn1(T65H)-mCherry, and dyn1(S61D)-mCherry showed similar recruitment signatures to scission and have similar enzymatic constants (see text). (D) Dyn1(T141A)-mCherry was similar to dyn1(WT)-mCherry, while dyn1(T141D)-mCherry showed significantly slowed recruitment kinetics at scission. (E) Dyn1(T65A)-mCherry was the least similar to all other recruitment signatures. Error bars represent standard error of the mean. (F) Dyn1(T65A)-mCherry was recruited from early time points at relatively high levels compared to dyn1(WT)-mCherry, other GTPase mutants, and GTPase effector domain (GED) mutants (dyn1(R725A)-mCherry, dyn1(K694E)-mCherry, dyn1(K694A)-mCherry). Fluorescence values calculated from window 82–42 s before scission (grey boxes, C–E). (G and H) Kymograph comparison of dyn1(WT)-mCherry and dyn1(T65A)-mCherry expressing cells illustrating conspicuous recruitment of dyn1(T65A)-mCherry to CCS from early time points. Red and yellow arrowheads indicate the timing of terminal and non-terminal scission event respectively. Blue asterisk marks the position of a CCS forming de novo.

We hypothesized that dynamin's GTPase activity was involved in recruiting dynamin to CCS. If this were correct, mutant versions of dynamin with altered enzymatic kinetics should have distinctive recruitment signatures relative to scission. To test this hypothesis we assayed the recruitment of six GTPase domain dynamin mutants [37],[38] tagged with mCherry (Figure 2A). The six GTPase mutations selected display a range of different K_m_ and k_cat_ values (see [37],[38]).

By inspection we found that GTPase domain point mutants with similar enzymatic kinetics had similar recruitment signatures. To quantify the similarity between the six GTPase mutants and wild type (WT), we compared the pre-scission recruitment signatures (from −82 s to t = 0) of each point mutant and WT pair-wise and arranged them into a dendrogram by hierarchal clustering (Figure 2B). Mutations that mainly effect k_cat_, dyn1(S61D)-mCherry, dyn1(T65D)-mCherry, and dyn1(T65H)-mCherry grouped together and slowed the burst of dynamin to a similar degree at time points close to scission (Figure 2C). Dyn1(T141A)-mCherry, a mutant with reduced affinity for GTP but greater rate of GTP hydrolysis, was surprisingly similar to dyn1(S61D/T65D/T65H) (Figure 2D). Dyn1(T141D), a mutant with reduced GTP binding and hydrolysis, had a dramatically slower rate of build-up in the seconds leading to scission (Figure 2D). Finally dyn1(T65A)-mCherry, a mutant with a low affinity for GTP and rate of GTP hydrolysis, was the least similar to any other recruitment signature, and had a unique recruitment profile (Figure 2E; see Video S1).

Dynamin has been implicated in regulating multiple stages of CCV formation [15]. To understand these regulatory mechanisms we focused on the dyn1(T65A) mutant, as its recruitment signature displayed a high level of recruitment from the earliest time points before scission (−80 s to −20 s pre-scission) (Figure 2E and 2F). This suggested dyn1(T65A) affected the transient “flickering” of dynamin and was recruited at higher levels to CCS during the early stages of CCS formation. Kymograph analysis of dyn1(WT)-expressing cells showed that dyn1(WT) was significantly recruited in the seconds before vesicle appearance (Figure 2G). In contrast dyn1(T65A) was significantly recruited at or shortly after the point of de novo CCS formation and appeared to be enriched at CCS from early time points (Figure 2H; and see Video S1).

In previous studies the expression of dyn1(T65A) was shown to significantly decrease the rate and extent of transferrin uptake measured using bulk assays [37]–[39], although it was not as potent a dominant negative as dyn1(K44A) [39]. Similar to these earlier results we found that dyn1(T65A) lowered the incidence of scission events; although, once again, it was a much less potent inhibitor of scission activity when compared to dyn1(K44A) (Table S1).

Unexpectedly, dyn1(T141D) did not cluster with dyn1(T65A) (Figure 2B), despite similar enzymatic constants [38]. Dyn1(T141D) did not affect the transient “flickering” phase of dynamin recruitment but had a slower burst of recruitment associated with scission, and hence was more similar to dyn1(S61D/T65D/T65H) (Figure 2B).

To further investigate the kinetics of dynamin association with CCSs we compared the mobility and turnover of dyn1(WT/T65A) using fluorescent recovery after photobleaching (FRAP). A 3-µm^2^ region containing CCS (indicated by mCherry-Clc) was selected and bleached and the fluorescence recovery of dyn1(WT/T65A)-EGFP and mCherry-Clc was measured in the following 100 s. Dyn1(T65A)-EGFP had a lower mobile fraction and slower half-time of recovery compared to dyn1(WT)-EGFP (mobile fraction of 59% versus 80%, and half time of recovery of 5.3 s versus 3.9 s, respectively) (see Figure S1A–S1F). Dynamin is recruited to clathrin-coated pits at the plasma membrane [12], and these results indicate that dyn1(T65A) turned over at CCS at a slower rate. The turnover and mobility, as measured by FRAP, of mCherry-Clc in cells expressing dyn1(WT) or dyn1(T65A) was very similar suggesting that the turnover of clathrin at CCS was independent of the dynamin GTPase cycle. These latter findings are congruent with published data [40].

Regardless of the mutant assayed using the pulse pH assay peak recruitment was at t = −2 s suggesting continuous dynamin recruitment up until the point of scission (see Table S1). All measurements were carefully performed at equivalent expression levels, although in a background of endogenous WT dyn1/2, and at present we do not know the relative proportions of endogenous and mutant dynamin at any given scission event. However, the variation between the recruitment profiles of specific mCherry-tagged GTPase mutants shows that dynamin's recruitment to CCS was regulated by its GTPase cycle.

### Dynamin GTPase Cycle Regulates Both CCS Maturation and Scission

**Figure 3:**
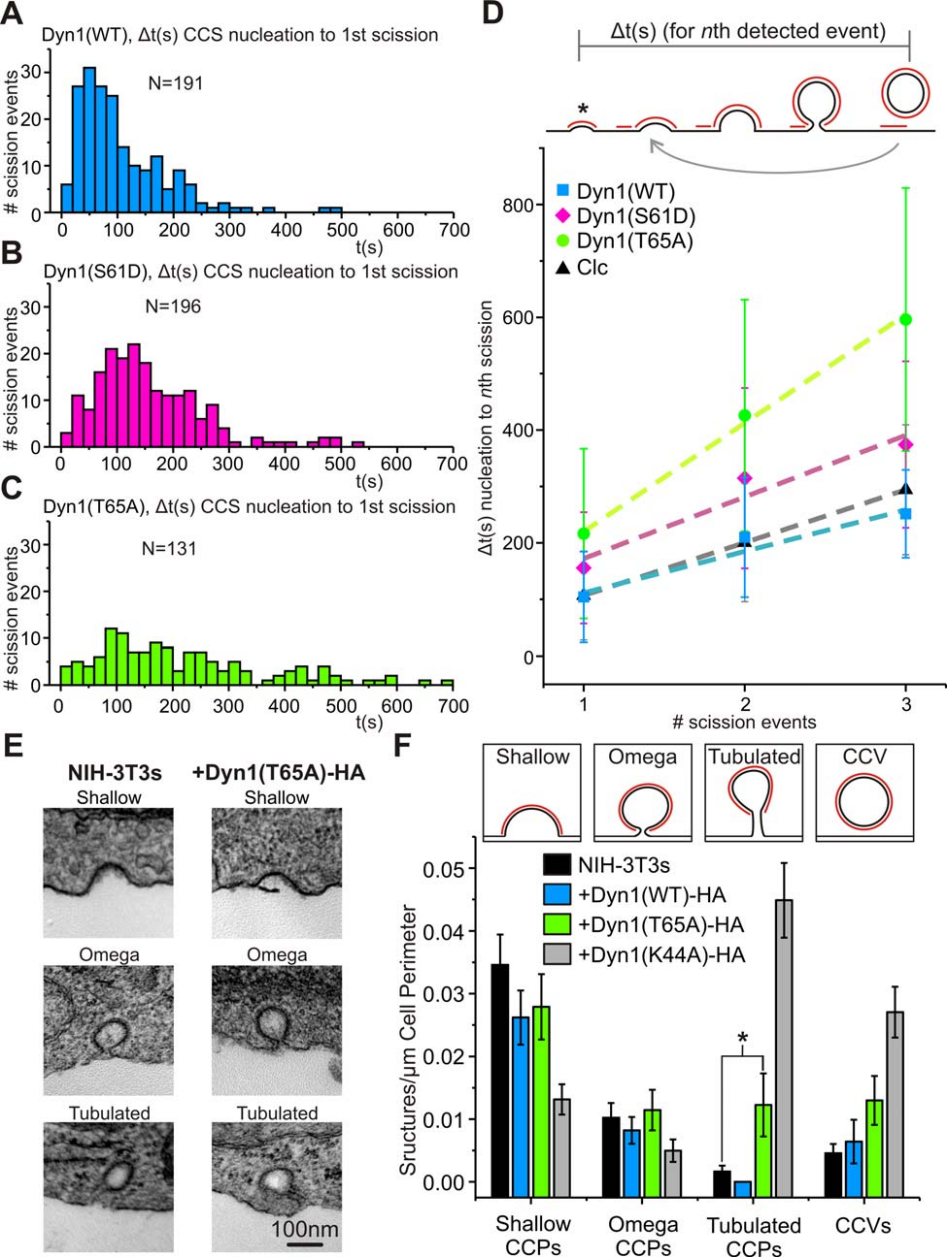
The effects of Dynamin GTPase mutants on CCS lifetime and ultrastructure. (A–C) Histograms of the time difference from de novo CCS nucleation to first detected scission events in cells expressing dyn1(WT)-mCherry (A), dyn1(S61D)-mCherry (B), and dyn1(T65A)-mCherry (C). (D) Slower rates of GTPase hydrolysis resulted in an increased average time from CCS nucleation to the *n*th scission event (see text for explanation of measurement). Expression of dyn1(T65A)-mCherry and dyn1(S61D)-mCherry increased the time from nucleation to the *n*th scission event compared to NIH-3T3 cells expressing dyn1(WT)-mCherry or clathrin light chain (Clc-mCherry). Dyn1(T65A)-mCherry had the most pronounced effect on CCS lifetime. (E) Ultrastructural analysis of coated pits in WT NIH-3T3 cells and cells expressing dyn1(T65A)-HA. Representative transmission electron micrographs of shallow, omega-shaped, and tubulated coated pits profiles as well as a coated vesicle. (F) Tubulated coated pits accumulate in cells expressing dyn1(T65A)-HA. Morphometric analysis of the frequency of coated pit profiles in NIH-3T3 cells, and in NIH-3T3 cells expressing dyn1(WT)-HA and dyn1(T65A)-HA. Insets show sketches of the coated pit profiles included in each category. Data were obtained from >30 randomly selected cell profiles. Error bars represent standard error of the mean. *p = 0.021, Student's *t* test.

Previous analysis of single scission events in 3T3 fibroblasts revealed CCS can host multiple scission events [26]. The time between CCS nucleation to the first detected scission event was equivalent to the time between successive scission events, if they occurred, at persistent CCS. Therefore the underlying kinetics of clathrin-coated bud formation was relatively constant, despite the observed variability of CCS lifetimes [33] and total CCS lifetime was not an accurate measure of the underlying kinetics of CCV formation for CCS, which hosted multiple scission events. To understand dynamin's role in CCS maturation, we measured the average time from CCS nucleation to the first detected scission event in cells expressing dyn1(WT), dyn1(S61D) and dyn1(T65A) (Figure 3A–3C). We found that expression of dyn1(S61D) and dyn1(T65A) increased the time from CCS nucleation to scission in comparison to the expression of dyn1(WT) (156 s and 216 s versus 104 s, respectively). For CCS, which hosted from one up to three scission events, the average time from nucleation to the *n*th scission event increased linearly, as expected (Figure 3D; see Materials and Methods). Therefore, dyn1(T65A) and dyn1(S61D) significantly slowed the underlying kinetics of clathrin-coated bud formation and, by definition, the endocytic activity per CCS was significantly lower in cells expressing these mutants. The magnitude of this effect was greatest for dyn1(T65A)-expressing cells. Dyn1(T65A) is a point mutant with a strong effect on GTP binding and hydrolysis, and its expression increased the time course of CCS maturation by ∼2-fold (Figure 3D).

For comparison the dynamics of three GED mutants were also measured: dyn1(K694A), dyn1(K694E), and dyn1(R725A) (see Figure S2A–S2C) [17],[18]. In earlier studies, it was suggested that dyn1(K694A) and dyn1(R725A) accelerated early, rate-limiting events in CCS maturation and thereby increased the rate of transferrin uptake [17],[18]. However, in an alternative study it was found cells expressing dyn1(K694A) and dyn1(R725A) internalized transferrin to an equivalent level as WT cells [37]. Our results were congruent with this later study and we found that the three GED mutants assayed did not have a strong effect on the dynamin recruitment signature (Figure S2A–S2C) or the average time from nucleation to scission (see Figure S2D–S2G).

To further investigate the strong effect of dyn1(T65A) on vesicle formation, we analyzed the ultrastructure of CCS in dyn1(WT), dyn1(T65A), and dyn1(K44A) expressing NIH-3T3 cells. Fluorescence-activated cell sorting was used to select NIH-3T3 cells expressing bicistronic constructs encoding dyn1(WT/T65A/K44A) tagged with hemagglutinin antigen (HA) and green fluorescent protein (see Materials and Methods). Cells were prepared for electron microscopy (EM) to investigate the effect of dynamin expression on the ultrastructure of coated pits. Compared to untransfected NIH-3T3s or dyn1(WT)-HA expressing NIH-3T3s, dyn1(T65A)-HA expressing cells accumulated coated pits with tubulated necks (Figure 3E and 3F; Student's *t* test *p* = 0.021); although the effect was not as strong as the expression of the dominant negative dyn1(K44A)-HA (Figure 3E and 3F). The potent effect of dyn1(K44A) on the incidence of scission (see Table S1) and the ∼4 fold increase in frequency of tubulated coated pits compared to cells expressing dyn1(T65)-HA (Figure 3F) suggested that the tubulated coated pits in the dyn1(K44A)-expressing cells were dead-end structures that were unable to pinch off, as previously concluded [12],[41]. By contrast the relatively moderate increase in tubulated coated pits in dyn1(T65A)-HA expressing cells suggested coated pits were slowed at this late phase close to scission, but were still endocytically competent structures that could pinch off to form CCVs. Collectively these data are consistent with previous studies and suggest dynamin's GTPase cycle regulates both clathrin-coated bud maturation and scission [14]–[19].

### Dynamin's GTPase Cycle Regulates the Kinetics of Actin and N-BAR Protein Recruitment

Dynamin directly interacts with F-actin [30], actin-associated proteins [42], as well as molecules that induce/sense membrane curvature such as N-BAR proteins [3],[21]. Given these biochemical interactions, how do the endocytic dynamics of actin and N-BAR proteins compare to that of dynamin? We observed transient foci of lifeact-mCherry, a vital probe for F-actin [43], at individual scission events that appeared ∼20 s before scission and then decayed in fluorescence intensity post-scission (Figure S3A). We observed endophilin2 (endo2)-mCherry recruitment (see Figure S3B) over a similar time window, consistent with previous studies [26] and localization of endo2-mCherry to the highly curved membrane neck of deeply invaginated CCS [44]. A comparison of the ensemble recruitment signatures confirmed that actin, endo2, and dynamin were recruited to sites of scission over a similar time window (Figure S3C and S3D; see also [26]). The ensemble recruitment signatures of NIH-3T3 cells stably expressing endo2-mCherry or transiently expressing BIN/Amphiphysin/RVS domain containing protein 1 (BIN1)-mCherry were very similar with a prominent burst of recruitment that peaked at 2–4 s before scission (Figure S3E; Table S1). These data suggest that actin, dynamin, and N-BAR proteins are present at CCS in the seconds leading up to scission and vesicle formation.

**Figure 4:**
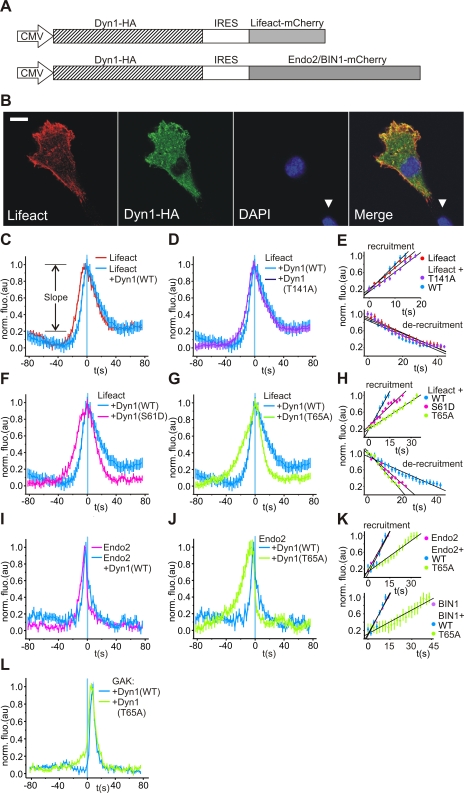
The effects of dynamin GTPase mutants on the kinetics of actin recruitment. (A) Bicistronic IRES vectors co-express dynamin tagged with HA epitope and lifeact-mCherry or endo2/BIN1-mCherry. (B) Immunofluorescence of NIH-3T3 cells transiently transfected with pIRESneoII-dyn1(WT)-lifeact-mCherry stained with antibodies against HA and mCherry. White arrowhead indicates untransfected cells only stained with DAPI (scale bar = 10 µm). (C) The fluorescence recruitment signature of lifeact-mCherry (red, six cells, 788 events) and lifeact-mCherry co-expressed with dyn1(WT)-HA (light blue, nine cells, 1,469 events). In cells co-expressing dyn1(WT)-HA, the recruitment peak of lifeact-mCherry was moderately shifted towards scission. Recruitment slope was measured over the interval shown. (D) The fluorescence recruitment signature of lifeact-mCherry co-expressed with dyn1(T141A)-HA (dark blue, nine cells, 2,258 events) compared with dyn1(WT)-HA (light blue). The recruitment signatures are very similar. (E) Comparison of the slope of lifeact-mCherry recruitment/de-recruitment in cells expressing lifeact-mCherry alone (magenta), co-expressed with dyn1(WT) (dark blue) or with dyn1(T141A) (light blue). The co-expression of dyn1(WT)-HA or dyn1(T141A)-HA had little effect on the slope of lifeact-mCherry recruitment/de-recruitment. (F and G) Expression of dynamin mutants with reduced rates of GTP binding and hydrolysis modified actin recruitment to sites of scission. Co-expression of dyn1(S61D)-HA (magenta, seven cell, 2,401 events) led to a left shift in lifeact-mCherry recruitment away from scission and a moderate decrease in slope compared to cells co-expressing dyn1(WT)-HA (blue) (F,H). Co-expression of dyn1(T65A)-HA (green, six cells, 797 events), the most potent GTPase mutant tested, led to a marked decrease in the slope of lifeact-mCherry recruitment compared to cells co-expressing dyn1(WT)-HA (blue) (G,H). The co-expression of dyn1(S61D)-HA or dyn1(T65A) increased the slope of de-recruitment post-scission compared to dyn1(WT)-HA control (H). (I–K) Expression of dynamin mutants with reduced rates of GTP binding and hydrolysis modified N-BAR recruitment to sites of scission. Endo2-mCherry recruitment kinetics were very similar in cells expressing endo2-mCherry alone (magenta, six cells, 2,608 events) and cells co-expressing dyn1(WT)-HA (blue, 12 cells, 2,186 events). (I,K) Co-expression of dyn1(T65A)-HA (green, seven cells, 574 events) led to a marked decrease in the slope of endo2-mCherry recruitment compared to cells co-expressing dyn1(WT)-HA (blue) (J,K). (L) The expression of dyn1(T65A)-HA had little effect on the kinetics of GAK-mCherry recruitment, a protein that is recruited post-scission and involved in the uncoating of CCVs.

Next we asked whether dynamin's GTPase cycle modulated actin and N-BAR protein recruitment to sites of scission. To examine if the kinetics of endocytic actin and N-BAR recruitment were regulated by dynamin's GTPase cycle we created bicistronic constructs to co-express lifeact-mCherry or endo2/BIN1-mCherry in tandem with WT or GTPase mutant versions of dynamin (Figure 4A and 4B). The recruitment signature of lifeact in a background of co-expressed dyn1(WT) and dyn1(T141A) showed only subtle differences to that of lifeact alone (Figure 4C and 4D) and had little effect on the slope of actin recruitment/de-recruitment (Figure 4E). Although it had no effect on the slope of actin recruitment, co-expression of dyn1(WT) did shift the lifeact recruitment signature ∼4 s towards scission (Figure 4C). The co-expression of dyn1(S61D) and dyn1(T65A) decreased the slope of actin recruitment with dyn1(T65A) having the strongest effect (Figure 4F–4H). Actin recruitment in cells expressing dyn1(S61D) had a shift in the recruitment signature ∼8 s away from scission. The actin recruitment signature in cells expressing dyn1(S61D) and dyn1(T65A) still peaked at t = 0, but F-actin build-up began at much earlier time points (>∼20 s and >∼30 s pre-scission for dyn1(S61D) and dyn1(T65A), respectively) (Figure 4H). In contrast the slope of actin de-recruitment from CCS was accelerated (Figure 4H). In all dynamin GTPase mutants assayed actin enrichment peaked at t = 0. We conclude from these experiments that the enrichment of actin at sites of CME scission was regulated by dynamin's GTPase activity either directly or indirectly and that dyn1(T65A), the most potent GTPase mutant tested, had the strongest effect.

The dynamin GTPase cycle had a similar effect on the rate of N-BAR protein recruitment to sites of scission. Expression of dyn1(WT) had no effect on the recruitment of endo2-mCherry to scission; however, the expression of dyn1(T65A) slowed the rate of endo2 recruitment to scission (Figure 4I–4K), although de-recruitment was the same. Expression of dyn1(T65A) had a similar effect on the kinetics of BIN1 recruitment (Figure 4K). However scission was still associated with peak N-BAR protein recruitment. We next examined how expression of dyn1(T65A) affected G-cyclin associated kinase (GAK) recruitment. GAK binds to Hsc70 and is involved in the uncoating reaction of CCVs [45]. We found that the expression of dyn1(T65A) had no effect on the rate of GAK-mCherry recruitment (Figure 4L) but the GAK recruitment profile was shifted 2 s towards scission (see Table S1). This could reflect an accelerated uncoating reaction following scission in cells expressing dyn1(T65A) (Figure 4H).

### Acute Inhibition of Actin Dynamics at Sites of Scission by the Addition of Latrunculin-B

**Figure 5:**
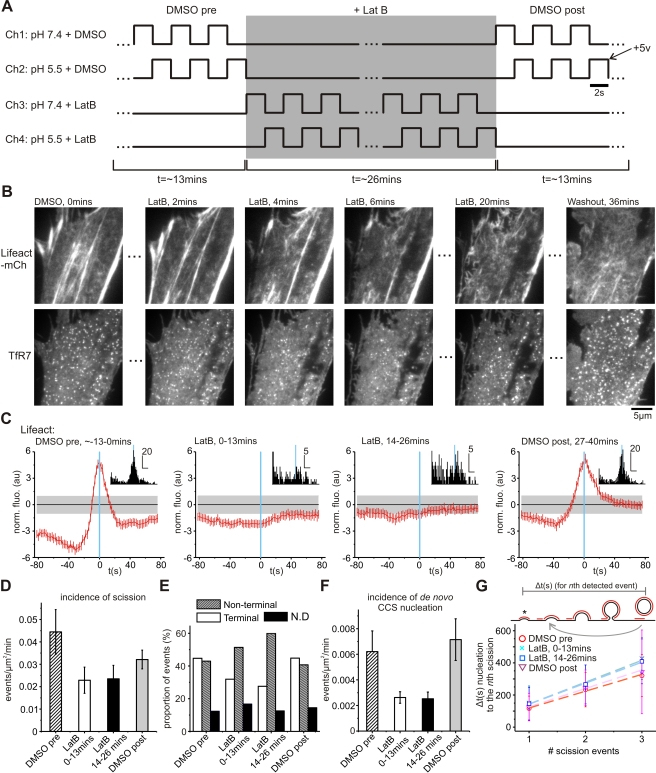
Acute ablation of actin recruitment to scission by addition of Latrunculin-B. (A) Logic diagram illustrating a four-channel perfusion system under transistor to transistor logic (TTL) control used to perfuse a target cell with drugs whilst detecting single scission events. Cells were first perfused with buffer containing vehicle (DMSO), then buffer containing latrunculin-B, followed by washout with buffer containing vehicle. (B) Example TIR-FM images of a cell expressing lifeact-mCherry and TfR-phl during acute exposure to 2.5 µM latrunculin-B. Within 6 min the majority of F-actin stress fibers had disappeared from the adherent cell surface and fewer CCS were visible. Washout of latrunculin-B resulted in the resumption of actin dynamics and a recovery in the number of CCS. (C) Lifeact-mCherry was not recruited to scission in the presence of latrunculin-B. Recruitment signature normalized to the random confidence interval (see Methods). Inset: histograms of peak lifeact-mCherry fluorescence (horizontal scale bar indicates the number of event; vertical scale bar corresponds to 20 s). Data pooled from five cells (DMSO pre-exposure, 1,559 events; 0–13 min exposure to latrunculin-B, 724 events; 14–26 min exposure, 758 events; and DMSO washout, 1,078 events). (D–F) Inhibition of actin dynamics reduced the incidence of scission by ∼50% (D), increased the proportion of scission events classified as non-terminal (E), and reduced the incidence of de novo CCS formation (F). Error bars represent SEM. (G) Inhibition of actin dynamics increased the time from de novo CCS nucleation to the *n*th detected scission event at CCS that host single or multiple scission events (limited to a maximum of three detected events). Error bars represent standard deviation.

Examinations of actin-disrupting drugs on CME have historically relied on pre-incubations (typically 30 min to 1 h) prior to examination with static techniques (such as biochemical assays or EM) or indirect optical assays for CME function [4],[7],[11],[46],[47]. Despite these studies the mechanistic role of actin in mammalian CME is still enigmatic. To gain a more precise mechanistic understanding of the role of actin in mammalian CME and how actin dynamics are interconnected with other endocytic proteins we developed a four-channel perfusion system, which allowed the local perfusion of a target cell with latrunculin-B in conjunction with the pulsed pH assay (Figure 5A). In a typical experiment a target cell was perfused with alternating high and low pH buffers containing vehicle DMSO (channel 1 and 2) for ∼13 min (400 frames) and then switched to alternating high and low pH buffer (channel 3 and 4) containing DMSO and 2.5 µM latrunculin-B. After 26 min (800 frames) the perfusate was switched back to channel 1 and 2 to wash out the latrunculin-B (Figure 5A). Image stacks were analyzed by separating them into 400-frame blocks corresponding to data acquired under DMSO, 0–13 min and 14–26 min exposure to latrunculin-B and during the wash out phase. Thus, unlike previous studies, we could directly measure CCS dynamics and scission events before, during, and after exposure to latrunculin-B.

On exposure to 2.5 µM latrunculin-B we observed the disappearance of actin foci, membrane ruffling, and actin stress fibers (Figure 5B). By 6 min lifeact-mCherry had a cytosolic localization, with only a few stable actin structures remaining (Figure 5B; see Video S2), and this was coincident with a reduction in the number of CCS (Figure 5B). In the presence of latrunculin-B we were unable to measure significant lifeact-mCherry recruitment relative to scission (Figure 5C). This finding is consistent with previous studies that have observed latrunculin-resistant clathrin endocytic events [7],[33],[47],[48]. Although scission events were observed, latrunculin-B reduced the incidence of scission by >50% (Figure 5D) and resulted in a greater proportion of events classified as non-terminal (Figure 5E). This suggested larger and more stable CCSs tended to host scission events in the absence of F-actin (see Table S1). Latrunculin-B decreased the incidence of de novo CCS formation (Figure 5F), consistent with previous studies [7],[11],[33]. Although the incidence of CCS nucleation was reduced in frequency, we measured an increase in the average time from nucleation to scission for CCS that did form de novo in the presence of latrunculin (150 s compared with 117 s in presence of DMSO only) (Figure 5G). Consistent with previous studies [7],[27],[49], ultrastructure analysis showed that latrunculin-B–treated cells accumulate omega shape pits with wider, less constricted membrane necks (see Figure S4A–S4C). On average the ratio of clathrin bud to neck diameter in coated pits classified as omega shaped was reduced by ∼one-third in NIH-3T3 cells treated with latrunculin-B (see Figure S4D). This suggested that actin was involved in membrane constriction and formation of deeply invaginated clathrin-coated pits.

### Actin Dynamics Regulate the Recruitment of Dynamin and N-BAR Proteins to Scission

**Figure 6:**
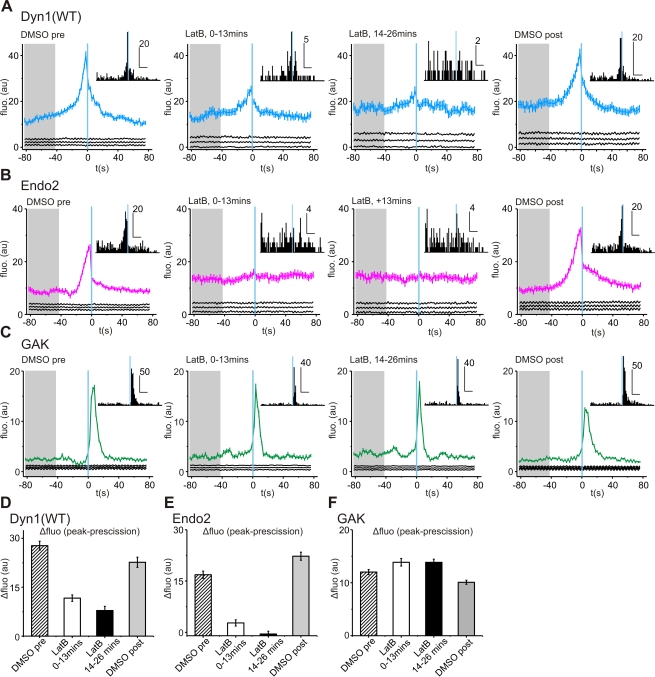
Inhibition of actin perturbs the kinetics of dynamin recruitment, but has minimal impact on GAK recruitment. (A) Acute inhibition of actin decreased the amplitude of peak dyn1(WT)-mCherry recruitment. Inset: histograms of peak dyn1(WT)-mCherry fluorescence for each scission event that composed the ensemble average (horizontal scale bar indicate the number of event, vertical scale bar correspond to 20 s). Recruitment traces from five cells: (DMSO pre-exposure, 875 events; 0–13 min exposure to latrunculin-B, 430 events; 14–26 min exposure, 451 events; and DMSO washout, 743 events). (B) The burst of endophilin2-mCherry recruitment at time points close to scission was ablated by exposure to latrunculin-B. Inset: histograms of peak fluorescence for each scission event. Recruitment traces of events from five cells (DMSO pre-exposure, 1,547 events; 0–13 min exposure to latrunculin-B, 885 events; 14–26 min exposure, 854 events; and DMSO washout, 1,445 events). (C) The kinetics of GAK recruitment was insensitive to latrunculin-B. Inset: histogram of peak fluorescence for each scission. Recruitment traces of events taken from three cells (DMSO pre-exposure, 1,070 events; 0–13 min exposure to latrunculin-B, 392 events; 14–26 min exposure, 472 events; and washout, 1,445 events). (D-F) Latrunculin-B exposure decreased the peak amplitude of dynamin (D) and endo2 (E) recruitment, but had a negligible effect on the amplitude of GAK recruitment (F). See Materials and Methods for details of quantification.

While a role for dynamin in regulating actin dynamics in cellular processes such as podosomes [50] and actin comets [28],[29] has been established, there are no data examining how actin regulates dynamin's endocytic function. We measured dynamin recruitment to sites of scission before, during, and after washout of latrunculin-B (Figure 6A). We found that latrunculin-B did not perturb the pre-scission “flickering” phase of dynamin recruitment but the burst of dynamin recruitment leading up to scission was strikingly decreased in amplitude (Figure 6A; see Video S3). Histogram analysis of dynamin fluorescence peaks revealed that between 0–13 min exposure to latrunculin-B there were still many events (∼16%) with a fluorescent peak at 2–4 s before scission (Figure 6A inset; see Materials and Methods) [26]. However after 13 min the fluorescent peaks appeared to be randomly distributed in time, indicating a reduction in the fluorescence intensity of the dynamin burst at scission relative to background fluorescence. These observations suggest that actin polymerization amplified dynamin recruitment at time points close to scission.

To test whether actin dynamics regulated the burst of dynamin at scission and not the pre-scission “flickering” at CCS, we examined the effects of latrunculin-B on the recruitment of the dyn1(T65A). The addition of latrunculin-B did not perturb the enrichment, or pre-recruitment, of dyn1(T65A)-mCherry at CCS (see Video S4). However, as observed with dyn1(WT), the disruption of actin dynamics with latrunculin-B decreased the amplitude of the final burst of dyn1(T65A)-mCherry recruitment at scission (Figure S5A and S5B). As observed in untransfected NIH-3T3s (Figure S4A), morphometric analysis of NIH-3T3 cells expressing dyn1(T65A)-HA exposed to latrunculin-B showed a significant increase in “omega” shape coated pit profiles and a reduction of “tubulated” coated pits (Figure S5C). Thus these data demonstrate that dynamin pre-scission flickering is actin independent and occurs at early stages during CCS maturation. By contrast the burst of dynamin at scission is actin dependent and correlates with the formation of a tubulated coated pit.

We next examined endo2 recruitment to scission events before, during, and after the washout of latrunculin-B. The addition of latrunculin-B caused the loss of endo2 puncta associated with CCS (see Video S5), consistent with previous studies [27]. In the presence of latrunculin-B the recruitment signature of endo2 was still above random; however, there was no longer a prominent burst of recruitment at time points close to scission (Figure 6B). Histogram analysis of the fluorescence peaks of endo2-mCherry at individual scission events were randomly distributed across time points before and after scission in the presence of latrunculin-B (Figure 6B, inset). When the perfusate was switched back to channel 1 and 2, endo2-mCherry recruitment to scission resumed with identical kinetics (Figure 6B; Video S5). Latrunculin-B had an identical effect on the BIN1 recruitment signature (unpublished data). We therefore conclude that although there was evidence of basal recruitment to CCS, a dynamic actin cytoskeleton was required for N-BAR recruitment to scission. Given that the recruitment profiles of BIN1 and endo2 were identical and responded in an identical manner to perturbations, we conclude that a single mechanism governs endo2 and BIN1 recruitment to CCS and that actin serves to concentrate N-BAR proteins at CCS at time points close to scission.

To examine whether inhibition of actin has an effect on CME reactions occurring post-scission, we examined the effect of latrunculin-B on the recruitment of GAK. We observed no difference in the magnitude or rate of GAK-mCherry recruitment in presence of latrunculin-B (Figure 6C; see Video S6). However, in the presence of latrunculin-B, peak GAK recruitment was shifted 4 s towards scission (see Table S1), suggesting that the post-scission de-polymerization of actin changed the timing of CCV uncoating relative to scission.

To quantify the effect that inhibition of actin dynamics had on the amplitude of dynamin and N-BAR recruitment we calculated the difference in fluorescence intensity between the early stages before scission (−82 to −42s pre-scission, grey area on graphs in Figure 6A and 6B) and peak recruitment relative to scission (see Table S1). Dynamin recruitment decreased by >50% on initial exposure to latrunculin-B (0–13 min) and >70% during 14–26 min exposure to latrunculin-B (Figure 6D). The effect on endo2 was even more dramatic with 14–26 min exposure to latrunculin-B ablating any enrichment of endo2 in comparison to pre-scission time points (Figure 6E). We conclude that changes in magnitude of the dynamin and N-BAR recruitment signal did not arise from a global repositioning of the plasma membrane or the clathrin bud in the evanescent field, because the amplitude of GAK recruitment was unaffected (Figure 6F).

## Discussion

Here we used quantitative microscopy to examine the kinetics of dyn1(WT) and dyn1(GTPase mutant) recruitment to individual endocytic events in live cells. Detailed analysis revealed two distinct phases of dyn(WT) recruitment: a low amplitude phase in the early stages of CCS maturation and a prominent spike that increased over 20–30 s and culminated in scission. Dynamin's GTPase cycle regulated both phases of recruitment, regulated the association of actin and N-BAR proteins with CCS and modulated CCS maturation. These experiments suggest that in addition to a mechanochemical enzyme dynamin also has a regulatory role, as proposed by previous studies [14]–[19].

The initial phase of dyn1(WT) association with CCS (<∼20–30 s prior to scission) manifested as low amplitude “flickering” (Figure 1), which was proportional to overall CCS size [26] and which suggested low copy number and most likely transient dynamin association with the clathrin lattice. The turnover of dynamin at CCS was confirmed using FRAP (Figure S1). Dynamin was localized to flat and hemispherical clathrin lattices using EM [12],[51] and a rate-limiting, mechanistic, role for dynamin at early time points has previously been reported [14],[15],[17]. The finding that dyn1(T65A) was enriched at early time points (Figure 2) suggested that dynamin's GTPase activity regulated the early recruitment phase. This finding was unexpected because, in the absence of highly curved membrane, dynamin was expected to be either unassembled or present as short-lived multimers with low instrinsic GTPase activity [52]–[55]. One possible explanation is that the low GTP affinity and rate of hydrolysis for dyn1(T65A) caused dynamin to be preferentially fixed in a conformation that stabilized linear oligomers [52],[53]. Alternatively the GTPase cycle could have regulated the binding kinetics of dynamin with SH3 domain interacting partners, such as intersectin [56], present at flat or hemispherical clathrin lattices [57].

Overall it seems unlikely that dynamin plays an essential role in the early stages of CCS formation because deeply invaginated tabulated coated pit profiles are still able to assemble in dynamin null fibroblasts [27]. However, dynamin is thought to be rate limiting [15],[17] and was shown to regulate the efficiency of these early CME reactions (Figure 3) [15]. Alternatively dynamin's transient association with CCS at early stages (where its activity is not required per se) could be a mechanism by which dynamin senses the progression or state of CCS maturation, possibly by interacting with SH3 domain containing proteins [20]. Such a mechanism could allow dynamin to be precisely targeted in the later stages of CCV biogenesis where it is mechanistically required for invagination and scission. This targeting could prevent coated pits from stalling, potentially leading to abortive endocytic events [15].

At ∼20–30 s before scission the dyn1(WT) fluorescent signal increased rapidly, which suggested a stabilized enrichment of dynamin at the CCS, which peaked at scission (Figure 1) [26]. This phase occurred over a similar time window as N-BAR protein and F-actin recruitment (Figure S3). Like the initial phase of dynamin recruitment, the kinetics and time course of this phase were regulated by its GTPase cycle (Figure 2). In addition, we showed that the expression of dynamin GTPase mutants with impaired GTPase activity (dyn1(S61D) and dyn1(T65A)) slowed the time course of N-BAR protein and actin polymerization over the ∼20–30 s leading up to scission (Figure 4H and 4I). This time course was similar to the later stages of invagination [33],[58] and suggested dynamin regulated the kinetics of invagination via the buildup of F-actin and N-BAR proteins.

**Figure 7:**
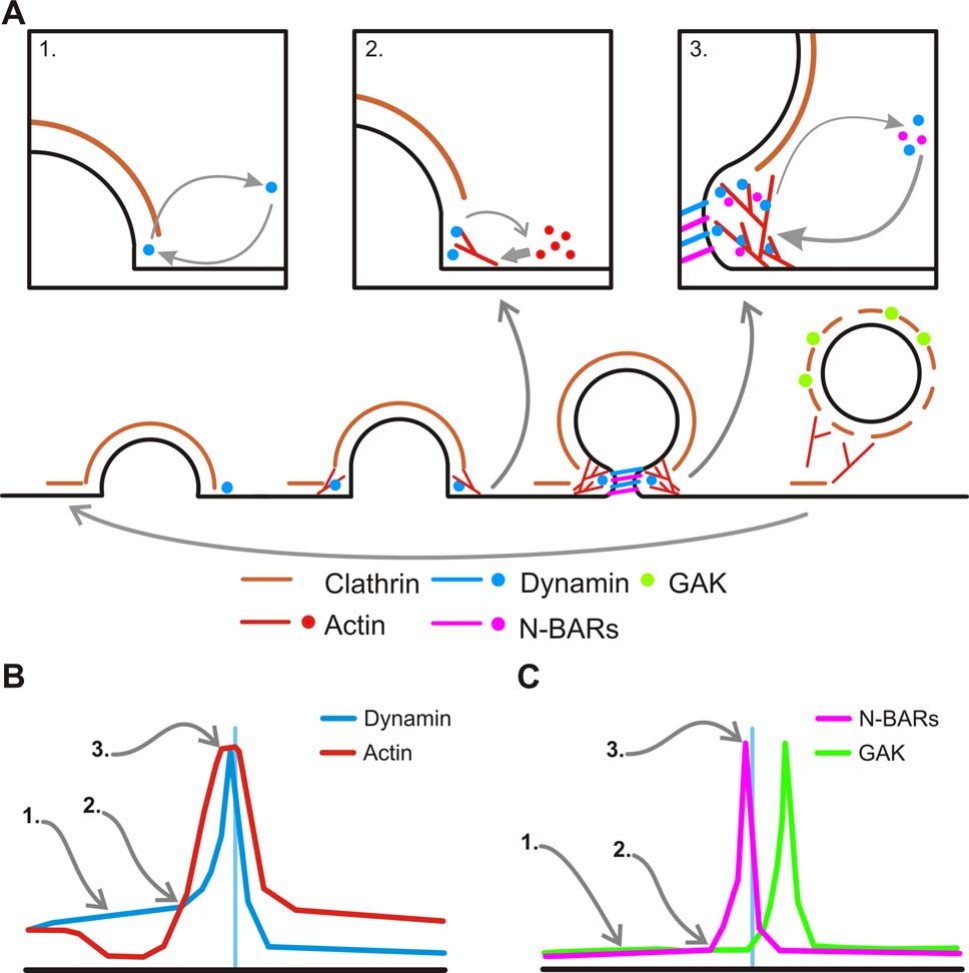
Model of the reciprocal regulatory mechanisms between dynamin and actin during CME. (A) During the early stages of CME dynamin has a transient association with the CCS which is modulated by the GTPase cycle (inset 1). As the CCS grows actin is recruited possibly generating auxiliary force to drive invagination. Dynamin regulates the rate of actin recruitment, in a mechanism that is dependent on it GTPase cycle (inset 2). The growth of dendritic actin networks around the clathrin bud form a scaffold that stabilizes dynamin and N-BAR proteins at the constricted neck of deeply invaginated CCS (inset 3). The coordinated recruitment of dynamin, actin, and N-BAR proteins promotes the efficient catalysis of membrane scission and the release the CCV into the lumen of the cell. The catalysis of scission also leads to the termination of actin polymerization and the breakdown the dendritic actin network. (B and C) Schematic of the dynamin/actin (B) and N-BAR/GAK recruitment profiles (C). We inferred (in absence of suitable measurements) where stages 1–3 (inset 1–3 from (A)) were relative to each profile.

Dynamin has a regulatory function in cellular processes dependent on Arp2/3-NWASP actin polymerization [28],[29],[42],[50]. However, does dynamin regulate actin polymerization directly or indirectly during CCV biogenesis? In an earlier study the analysis of arrested coated bud profiles in dynamin1/2 knock out fibroblasts suggested that dynamin terminated actin dynamics at CCS by mechanically catalyzing scission [59]. The increased frequency of tubulated coated pits in cells expressing dyn1(T65A) (Figure 3F) is congruent with this hypothesis. However, the subtle effects that distinct GTPase mutants have on the rate of lifeact recruitment suggest a direct regulatory role on the kinetics of F-actin polymerization at CCS. Whether dynamin exerts its effect on actin dynamics via endocytic actin effectors such as syndapin [60], cortactin [31],[61], or Abp1 [32], via a second GTPase Cdc42 [62] and/or more directly by binding F-actin and promoting uncapping [30], remains unclear. Nonetheless we believe our data are congruent with the idea that dynamin's regulatory role on actin dynamics during CME has a temporal context [15] and that dynamin can function as a regulatory GTPase, which controls actin polymerization, and a mechano-chemical enzyme, but that these functions are temporally distinct (Figure 7) [14],[16]–[19].

The role of actin in mammalian CME is controversial. As we have shown here productive CME scission events do occur in the presence of actin-disrupting drugs, similar to previous results [7],[33],[46]. These observations have led to the suggestion that actin is dispensable in the canonical mammalian model of CME [1], at least for cultured cells. Nonetheless, disrupting the actin cytoskeleton clearly has profound effects on the CME machinery: the efficiency of the entire reaction was significantly impaired (Figure 5) including, from morphological analysis, the final stages of CCS invagination (see Figure S4). Moreover, we along with others [7],[26],[27],[33],[63] have observed that actin and actin-associated factors are recruited and enriched at CCS in mammalian cells. We now show that actin modulates the degree of recruitment of N-BAR proteins and dynamin. Collectively these data suggest that evolution has indeed delegated a functional role for actin in mammalian CME. Perhaps a more pertinent question for future studies is why in mammalian cells, in comparison to yeast, CME has evolved a high level of mechanistic redundancy.

The detailed debate over the potential function of the actin cytoskeleton in CME has primarily focused on whether actin generates the force for membrane deformation during invagination [64]–[67] or plays a role in lipid segregation [68]. Our experiments did not disprove these potential functions. However, we showed that a dynamic actin cytoskeleton did control the amplitude of dynamin and N-BAR recruitment to the CME scission reaction (Figure 6). Thus, in addition to a mechanical function, we suggest actin serves as a scaffold to concentrate endocytic effectors at CCS (Figure 7), perhaps similar to mechanisms found in neurons [69]. The decrease in amplitude of the dynamin signal in the presence of latrunculin-B suggests that, under non-perturbed conditions, a greater amount of dynamin was recruited to CCS than was required to catalyze scission. Under normal conditions an actin scaffold could stabilize dynamin above the minimum threshold required for scission, promoting dynamin assembly and a more efficient scission reaction (Figure 7).

Structural studies on dynamin have proposed that membrane scission is driven by GTP binding and dimerization between adjacent rungs in the dynamin spiral [52],[70]. This event results in the formation of a “productive” dynamin spiral that can exert shear forces on the membrane when GTP is hydrolyzed [52], thereby leading to the catalysis of membrane scission. The slower build up of GTPase mutants in the ∼20–30 s preceding scission (Figure 2) and the prolonged lifetime of CCS in dyn1(S61D/T65A) expressing cells (Figure 3) could partially be a result of the slower formation of a “productive” dynamin spiral. However we believe that subtle rearrangements of assembled dynamin at the deeply invaginated coated pit [52],[70] cannot solely account for the longer time course of dyn1(S61DT65A) recruitment. Instead a more likely scenario is the slower build up of dynamin GTPase mutants reflected a slower rate of invagination and neck constriction.

Events immediately following scission were subtly affected by both dyn1(S61D/T65A) expression and latrunculin B treatment. In cells expressing dyn1(S61D/T65A) the dissociation of actin and N-BAR proteins from sites of scission was accelerated (Figure 4), while in cells transiently bathed in latrunculin B the post-scission accumulation of GAK was slightly augmented (Figure 6F). This finding suggests that some factor necessary for events following scission, perhaps a particular phosphoinositide [71], accumulates in nascent clathrin-coated buds with an impaired scission machinery.

Collectively our data are congruent with a model wherein dynamin's association with CCS follows a series of temporally distinct stages (Figure 7). Association of dynamin with coated pits at stage 1 (<∼20–30 s prior to scission) most likely requires the proline rich domain (PRD) [39] and the amount of dynamin recruited is proportional to the size of the clathrin lattice [26]. The accelerating accumulation of dynamin in the late stages of invagination in stage 2, 20–30 s prior to scission, is augmented by actin and, in turn, dynamin controls the rate of actin accumulation and the recruitment of curvature inducing/sensing N-BAR proteins. This forms a feedback loop, making the late stages of invagination directional and essentially irreversible. Stage 3 is scission itself at the deeply invaginated CCS, mediated by dynamin [23],[36].

The findings presented here show that, in addition to its well-established role in scission (stage 3), dynamin's GTPase cycle is also involved in the targeting of dynamin to CCS in stages1 and 2 and modulates the recruitment of actin and N-BAR proteins in the late stages of invagination (stage 2). The major challenges are to unravel the mechanism(s) by which dynamin's GTPase activity controls dynamin targeting to CCS prior to the formation of a constricted invagination, to precisely understand how dynamin controls actin polymerization in the final stages of invagination and to correlate the observed patterns of protein recruitment to dynamic changes in CCS topology.

## Materials and Methods

### Cell Culture, DNA Plasmids, and Constructs

NIH-3T3s were maintained in DMEM with 5% calf serum (Invitrogen). NIH-3T3 fibroblasts were maintained at 37°C and 10% CO_2_. Cells were transfected with lipofectamine 2000 according to the manufacturer's instructions (Invitrogen). Stable cell lines expressing mouse dynamin1-mCherry (Addgene plasmid: 27697), or rat endo2-mCherry (a generous gift from the De Camilli lab, and previously described [72]) were generated by selection with media containing 350 µg/ml G418 (Invitrogen).

Mouse dynamin1 point mutants were made using Quicktime site directed mutagenesis kit as per the manufacturer's instructions (Stratagene). The following primers were used to generate dynamin point mutants: dyn1(K44A): forward 5′: gtggtaggcggccagagcgccggcgcgagctcggtgctggagaatttcgtg, reverse 5′: cacgaaattctccagcaccgagctcgcgccggcgctctggccgcctaccac; dyn1(T65A): forward 5′: ggatctggcatcgtcgcccggcgtcccctggtc, reverse 5′: gaccaggggacgccgggcgacgatgccagatcc; dyn1(S61D): forward 5′: gacttcttgccccgaggagatggcatcgtcacccggcg, reverse 5′: ccgggtgacgatgccatctcctcggggcaagaag; dyn1(T65D): forward 5′: ggatctggcatcgtcgaccggcgtcccctggtc, reverse 5′: gaccaggggacgccggtcgacgatgccagatcc; dyn1(T65H) forward 5′: ggatctggcatcgtccaccggcgtcccctggtc, reverse 5′: gaccaggggacgccggtggacgatgccagatcc; dyn1(T141A): forward 5′: gacctgccaggaatggccaaggtcccagttggg, reverse 5′: cccaactgggaccttggccattcctggcaggtc; dyn1(T141D): forward 5′: gacctgccaggaatggacaaggtcccagttggg, reverse 5′: cccaactgggaccttgtccattcctggcaggtc; dyn1(K694A): forward 5′: ctcatgatcaacaacaccgcggagtttatcttctctgag, reverse 5′: ctcagagccgataaactcgatggtgttgttgatcatgag; dyn1(K694E): forward 5′: ctcatgatcaacaacaccatcgagtttatcttctctgag, reverse 5′: ctcagagaagataaactcgatggtgttgttgatcatgag; dyn1(R725A): forward 5′: gccgagcaggctcagcgggccgacgagatgctgcgcatg, reverse 5′: catgcgcagcatctcgtcggcccgctgagcctgctcggc.

Mouse dyn1(WT/K44A/S61D/T65A/T141A) was engineered with a N-terminal HA tag by PCR and ligated into the pIRESneoII-EGFP vector (Clontech) using Bgl2 and EcoR1 restriction sites (forward primer 5′: gcgcgcagatctaccatgggcaaccgcggcatggaa; reverse primer 5′: gcgcgcgaattcctaagcgtaatctggaacatcgtatgggtaacttccactggggtcactgatagtgattct). To generate pIRESneoII-Dyn1-HA(WT/K44A/S61D/T65A) the EGFP open reading frame was removed by digest with BstX1 and Not1. Lifeact-mCherry was engineered to include BstX1 and Not1 sites by PCR (forward primer 5′: agcttaccatgggagtggcggacctcatcaagaagttcgagagtatcagtaaggaggagctgca; reverse primer 5′: gctcctccttactgatactctcgaacttcttgatgaggtccgccactcccatggta) and digested and ligated into BstX1/Not1 digested pIRESneoII-EGFP vector. To construct pIRESneoII-Dyn1-HA-GAK-mCherry and pIRESneoII-Dyn1-HA-endo2/BIN1-mCherry a second multi-cloning site (forward 5′: ttggacgcgtttcgtacggagctcctgcaggctcgacctgcagcgc) was constructed downstream of the IRES sequence by removing the EGFP opening reading frame by restriction digest with BstX1/Not1. This generated a unique Mlu1 and BsiW1 site. GAK-mCherry (Addgene plasmid: 27695) was amplified by PCR (forward primer 5′: gcgcgcacgcgtaccatgtcgctgctgcagtctgcg; reverse primer 5′: gcgcgccgtacgttacttgtacagctcgtccat), engineered to include Mlu1 and BsiW1 sites, digested and ligated into the second MCS to generate pIRESneoII-Dyn1HA-GAK-mCherry. Similarly BIN1-mCherry (Addgene plasmid: 27693) and endo2-mCherry were amplified with Mlu1 and BsiW1 ends (BIN1 forward primer 5′: gcgcgcacgcgtaccatggcagagatcgggagc; endo2 forward primer 5′: gcgcacgcgtaccatgtcggtggcggggctg; reverse primer was the same as used to amplify GAK-mCherry), digested and ligated into the MCS to generate pIRESneoII-Dyn1HA-BIN1/endo2-mCherry. All constructs used were verified by DNA sequencing.

### Total Internal Reflection Microscopy and Perfusion

The TIR-FM microscope and pulsed pH assay for detecting endocytic scission events has been described previously [26]. Briefly, the TfR was tagged with pH sensitive super-ecliptic phluorin to generate TfR-phl [26],[33]. TIR-FM image series were acquired in synchrony with alternating pH of 7.4 and pH 5.5. Scission events manifested as the abrupt appearance of pH insulated spots in images acquired at pH 5.5 [26],[33]. To combine the pulsed pH assay with the wash-on/wash-off of drugs, such as latrunculin-B (Calbiochem), a four-channel perfusion system was built in house. In a typical experiment 400 frames (∼13 min) were acquired as the pH was switched between pH 5.5 and pH 7.4 with buffers containing DMSO (0.04% v/v). After 400 frames, perfusion was switched to pH 5.5 and pH 7.4 buffers containing DMSO (0.04% v/v) and 2.5 µM latrunculin-B. In a typical experiment ≥800 frames (∼26 min) were acquired in presence of latrunculin-B. After 1,200 frames (∼39 min) perfusion was switched back to channels containing DMSO for a further 400 frames (∼13 min) wash-out. For analysis the image series was divided into 400 frame blocks consisting of data acquired pre-exposure, 0–13 and 14–26 min exposure to latrunculin-B, and post-exposure washout. Data were analyzed as described below.

### Image Analysis of Pulse pH Data

In the following a TfR5 spot refers to a pH-resistant TfR-phl spot in an image acquired at pH 5.5 and a TfR7 spot refers to a spot or blob of TfR-phl, concentrated at a coated pit, in an image acquired at pH 7.4. All data acquired with the pulse pH assay were analyzed as previously described [26],[33]. Briefly, image series acquired with alternating pH were de-interlaced into pH 7.4 and pH 5.5 image series. Candidate scission events manifested as the abrupt appearance of TfR5 spots that were identified by multi-particle tracking using the MIA applet (Multidimensional Image Analysis, V. Racine and J.B. Sibarita, Curie Institute, Paris, France) for Metamorph (Molecular Devices). Candidate TfR5 scission events were screened for S/N, persistence, and fluorescence change as described previously [26],[33]. All fluorescence data were quantified using Matlab (Mathworks) and data stored in Excel (Microsoft). Previous work established that: (1) TfR7 spot fluorescence is highly correlated with the amount of clathrin at a CCS and faithfully report CCS dynamics and (2) that CCS can host multiple scission events [26]. Therefore, to measure the time from CCS nucleation to the *n*th scission event TfR7 spots were tracked and those TfR7 track histories, which: (1) formed de novo during image acquisition and (2) were associated with one or more bonafide scission events were isolated and manually verified for TfR7 tracking fidelity. Broken track histories were edited and fixed if necessary. For each verified de novo TfR7 track history the timing of associated scission events was noted and recorded to Excel workbooks (Microsoft). The time between TfR7 spot formation and the *n*th scission event was then calculated.

### Cluster Analysis and Dendrogram of Dynamin GTPase Mutants

The average recruitment signatures of mCherry-tagged dynamin GTPase point mutants were compared by computing the correlation coefficients for each pair of curves corr(dyn1(XX)_a_,dyn1(YY)_b_). Cosine distances were used to calculate the correlation coefficients between the mCherry recruitment curves as this transform was deemed more sensitive to shape. To focus on the shape of the recruitment curve prior to scission only time points between −82 s and 0 s were used for cluster analysis. Hierarchal clustering was performed using an average linking algorithm and represented by a dendrogram. The final dendrogram had a cophenetic correlation coefficient of 0.83 and other linkage algorithms yielded lower correlation coefficients. All calculations and construction of the dendrograms were performed in Matlab (Mathworks).

### Electron Microscopy

NIH-3T3 cells transfected with pIRESneoII-Dyn1-HA(WT/K44A/T65A)-EGFP were sorted away from untransfected cells by FACS. They were then re-plated and allowed to grow overnight to recover. For electron microscopy of resin-embedded sections, cells grown in Petri dishes were briefly washed twice with PBS and then fixed in paraformaldehyde (PFA; 2%) and glutaraldehyde (2.5%) in sodium cacodylate (0.1 M at pH 7.2). To examine CCS ultrastructure in the presence of latrunculin-B NIH-3T3s cell were incubated for 10 min in HBS containing either latrunculin-B (2.5 µM) or DMSO. Cells were then washed and fixed as described above. Post-fixation the cells were gently scraped off and centrifuged in a horizontal rotor. The cell pellet was then placed in fresh fixative and stored at 4°C. Samples were washed thoroughly in sodium cacodylate buffer (0.1 M) and post fixed in OsO4 (1% in 0.1 M sodium cacodylate) for 1 h and then washed with distilled water (both OsO4 and cacodylate buffer purchased from Agar Scientific). Samples were stained en bloc with uranyl acetate (2%) in ethanol (30%) before dehydration in a graded ethanol series followed by 1,2, epoxy propane (propylene oxide) and then infiltrated and embedded in CY212 resin (Agar Scientific). Ultrathin (50–70-nm) sections were cut on a Reichert Ultracut E microtome (Leica) and collected on uncoated 200 mesh grids. Sections were post-stained with saturated uranyl acetate before staining with Reynolds lead citrate [73]. Images were acquired using a Philips EM208 microscope (Philips), with an operating voltage of 80 kV, and a CCD camera detector. For morphometric quantification intact cell profiles were selected at low magnification (typically 3,500×–4,400×) at which coated pit structures were not visible. A low power image was acquired to measure the cell perimeter. Cells were selected on the basis of an intact cell perimeter to ensure the quantification of coated structures remained blind. Subsequent high power images were acquired on clathrin-coated structures with 200 nm of the plasma membrane for each intact cell selected. Cell perimeter was estimated by counting intersections between a 1 µm over laid lattice and the plasma membrane [74],[75]. Omega-shaped and tabulated coated pits were distinguished from shallow coated pits by the presence of inward curvature. Coated structures scored as coated vesicle likely corresponded to a glancing section through shallow, omega, or tabulated structures [76]. To determine the ratio of omega-coated pit bud to neck diameter high magnification (71,000×) images of omega pit profiles were taken. Measurements of diameter were made in ImageJ and exported to Microsoft Excel for subsequent analysis.

### Immunofluorescence

Cells were seeded onto coverslips 12–24 h before being fixed with a solution of 4% PFA in PBS for 5 min at room temperature. The coverslips were then washed in a solution of 0.05% TritonX-100 in PBS for 2 min to permeabilize the cells. The coverslips were rinsed in PBS and the primary antibodies were added diluted in PBS with 0.1% BSA. The primary antibodies used were mouse anti-HA (Invitrogen) and rabbit anti-RFP (Abcam). The coverslips were incubated with primary antibodies overnight. Coverslips were washed in PBS with 1% BSA and transferred to secondary antibodies (donkey anti-mouse-Alexa488 and goat anti-Rabbit-Alexa568) diluted in PBS with 0.1% BSA. After incubation for 1 h, the coverslips were rinsed in PBS and mounted on slides with Vectorshield (Vector laboratories) containing DAPI to stain the nucleus. Samples were imaged using a 510 Zeiss confocal laser scanning microscope using a 63×/1.4 NA oil immersion Plan-Apochromat Zeiss lens.

### Statistical Analysis

A two-tailed Student's *t* test in Microsoft Excel was carried out on EM data to determine statistical differences.

### FRAP Microscopy and Analysis

NIH-3T3 cells were transfected with dyn1(WT/T65)-EGFP and mCherry-Clc (Addgene plasmid: 27680) using Lipofetamine2000 (Invitrogen) and plated onto Lab-Tek chamber coverslips (Thermo Fischer Scientific) to adhere overnight. 24 h post-transfection cells were placed in HEPES buffered saline imaging buffer. FRAP experiments were performed on an Nikon TiE inverted microscope (Nikon Instruments) equipped with a Andor Revolution Spinning Disk confocal microscopy system and a Andor FRAPPA scan head (Andor Technology). FRAP experiments were performed on a heated stage (Oko labs, Naples Italy) set a 30°C, to be consistent with measurements made using the pulse pH assay and TIR-FM. Target cells were imaged with a 100× 1.4NA objective lens and sequentially illuminated with 488 nm and 561 nm laser light at a frame rate of 1 Hz with a exposure time of ∼450 ms per frames. Images were captured on iXon3 897 EMCCD camera (Andor Technology) with a pixel size 0.13 µm. A 3-µm^2^ region was selected for FRAP and subjected to 100% laser power (20 m W laser) scan with 20 ms dwell time per pixel with five repeats of the selected region. Ten frames were collected before photo-bleaching, and 100 frames were collected after bleaching to analyze fluorescent recovery. FRAP data were analyzed using Andor iQ software (Andor Technology). The raw fluorescent values were corrected for bleaching using an equivalent sized reference region in an un-bleached portion of the cell. An inverse exponential decay, was fit to the post-bleach fluorescence recovery data (e.g., *F*(*t*) = 1−*Ae* ^−Kt^). The mobile fraction was calculated from pre-bleach fluorescence intensity (*I*_0_) and the post-bleach fluorescence intensity (*I*_t_) following recovery (e.g., m mobile fraction % = *I*_t_/*I*_0_). The half-time of recovery was calculated from the fitted parameters *K* (e.g., half time = *ln*(0.5)/−*K*).

## Supporting Information

Figure S1 **The turnover and mobility of Dyn1(WT/T65A)-eGFP and mCherry-Clc analyzed by FRAP.** (A and B) An NIH-3T3 cell co-transfected with dyn1(WT)-EGFP (A) and mCherry-Clc (B). The mobility and turnover of dyn1 and Clc was analyzed using spinning disk confocal and FRAP. A 3-µm^2^ region (white box) containing CCSs was selected and bleached. The recovery of the fluorescent signal was analyzed to determine the mobile fraction and half time of recovery of dyn1(WT) and Clc (fluorescent time course in (A) and (B), see Methods for details of analysis). (C and D) NIH-3T3 cells co-transfected with dyn1(T65A)-EGFP (C) and mCherry-Clc (D) analyzed with FRAP (fluorescent time course in (C) and (D)). (E) The average mobile fraction, as determined by FRAP, of dyn1(WT) (*n* = 20 cells) and dyn1(T65A) (*n* = 17 cells). On average Dyn1(T65A) had a decreased mobile fraction in comparison to dyn1(WT) (59% versus 80% mobile fraction, respectively; Student's *t* test *p*<0.001) suggesting a significantly increased association with CCS. The mobility of mCherry-Clc was also analyzed and found not to be significantly different in cells expressing dyn1(WT) or dyn1(T65A) (53% versus 51% mobile fraction, respectively; Student's *t* test *p* = 0.61). (F) The average half time of fluorescence recovery for dyn1(WT) and dyn1(T65A). Dyn1(T65A) had a slower recovery time than dyn1(WT) (5.3 s versus 3.9 s, respectively; Student's *t* test *p* = 0.027). The recovery time of mCherry-Clc was not found to be significantly different in cells expressing dyn1(WT) or dyn1(T65A) (13.8 s versus 12.4 s, respectively; Student's *t* test *p* = 0.31). Error bars represent standard error of the mean (SEM).
(TIF)

Figure S2 **The effects of dynamin GED domain mutants on dynamin recruitment kinetics and de novo CCS lifetime.** (A–C) Comparison of dyn1(WT)-mCherry and GED domain mutant recruitment signatures. The recruitment signatures of dyn1(K694A)-mCherry (A), dyn1(K694E)-mCherry (B), and dyn1(R725A)-mCherry (C) displayed similar recruitment kinetics to scission as dyn1(WT)-mCherry. (D–F) Histograms of the time difference from de novo CCS nucleation to first detected scission events in cells expressing dyn1(K694A)-mCherry (D), dyn1(K694E)-mCherry (E), and dyn1(R725A)-mCherry (F). (G) Point mutations in the GED region of dynamin had little effect upon the average time from CCS nucleation to the *n*th scission event (see text for explanation of measurement). Expression of dyn1(K694A/K694E/R725A)-mCherry did not significantly increase the time from nucleation to the *n*th scission event compared to NIH-3T3 cells expressing dyn1(WT)-mCherry. Error bars represent standard deviation.
(TIF)

Figure S3 **Actin and N-BAR proteins were recruited at the same time as Dynamin to sites of scission but with distinct kinetics.** (A) Actin was recruited in the moments leading up to vesicle appearance. (Ai) A time series of an example scission event in a cell expressing lifeact-mCherry showing the recruitment of F-actin to sites of scission. (Aii) Fluorescent measurements (dark green, TfR7; light green, TfR5; red, lifeact) of the event shown in (Ai). Dots correspond to the images shown in (Ai). (B) The N-BAR protein endo2 was recruited in the seconds preceding vesicle appearance. (Bi) Time series of an example scission event in an NIH-3T3 cell expressing endo2-mCherry. (Bii) Fluorescence measurements from the time series shown in (Ai) (dark green, TfR7; light green, TfR5; purple, endo2). Dots correspond to the time series shown. Horizontal scale bars in (A) and (B) corresponds to 20 s, and blue line represents t = 0, the moment of scission. (C) Ensemble recruitment signature of lifeact-mCherry (6 cells, 788 events) compared to dyn1(WT) (11 cells 3157 events). Lifeact recruitment was measured in NIH-3T3 cells transiently expressing lifeact-mCherry (D). Comparison of endo2-mCherry and dyn1(WT)-mCherry recruitment signatures. Endo2-mCherry recruitment was measured in NIH-3T3 cells stably (St) expressing endo2-mCherry (6 cells, 2608 events). (E) N-BAR proteins BIN1 and endo2 showed similar recruitment kinetics to scission. The normalized recruitment signature from NIH-3T3 cells stably expressing endo2-mCherry and transiently (Tr) expressing BIN1-mCherry (11 cells, 3,024).
(TIF)

Figure S4 **Ultrastructure of CCS in the presence of latrunculin B.** (A) Morphometric analysis of coated pit profiles in NIH-3T3 exposed to DMSO or latrunculin B. Insets show sketches of the coated pit profiles included in each category. Data were obtained from >30 randomly selected cell profiles. Error bars represent standard error of the mean. (B and C) Example images of omega-shaped coat pit profile from NIH-3T3 exposed to DMSO or latrunculin B. Omega-shaped coated pits in cell exposed to latrunculin B appear to have wider necks. (D) Quantification of the ratio between the bud and neck diameter of omega-coated pits suggest an inhibition of neck constriction in the presence of latrunculin B. Measurement made from a subset of >30 structures classified as omega-coated pits. Error bars represent standard error of the mean. Student's *t* test, *p* = 0.0097.
(TIF)

Figure S5 **Actin remodeling is not required for the concentration of dyn(T65A)-mCherry at CCSs.** (A) Acute inhibition of actin decreased the amplitude of peak dyn1(T65A)-mCherry recruitment at scission, but does not perturb the pre-scission recruitment. Inset: histograms of peak dyn1(T65A)-mCherry fluorescence for each scission event that composed the ensemble average (horizontal scale bar indicate the number of event, vertical scale bar correspond to 20 s). Recruitment traces from five cells: (DMSO pre-exposure: 383 events, 0–13 min exposure to latrunculin-B; 116 events, 14–26 min exposure; 178 events, and DMSO washout: 342 events). (B) Latrunculin-B exposure decreased the peak amplitude of dyn1(T65A), in a similar manner to dyn1(WT) (see Figure 6). See Materials and Methods for details of quantification (C). Morphometric analysis of coated pit profiles in NIH-3T3 expressing dyn1(T65A)-HA exposed to DMSO or latrunculin B. As with untransfected NIH-3T3 cells (see Figure S4), treatment with latrunculin-B resulted in an increase in the frequency of “omega”-shaped coated pit profiles. Insets show sketches of the coated pit profiles included in each category. Data were obtained from >30 randomly selected cell profiles. Error bars represent standard error of the mean.
(TIF)

Table S1 **Parameters for the cells used in this study.** Expression: whether the construct was transiently or stably expressed. #cells, number of cells recorded; #events, total number of events detected and analyzed; #Term, number of events classified as terminal, i.e., with disappearance of the CCS (see Methods); #Nterm, number of events classified as non-terminal (no CCS disappearance); %Term, percentage of terminal events; Peak time, time of maximum average fluorescence relative to CCV detection, in seconds; Scission rate, average over individual cells of the rate of event detection per µm^2^ per minute, error represents standard error of the mean.
(JPG)

Video S1 **Comparison of dyn1(WT), dyn1(T65A), and TfR7 dynamics in NIH-3T3 cells.** NIH-3T3 cell transiently expressing hTfR-phl and dyn1(T65A)mCherry shown on top. NIH-3T3 cells transfected with hTfR-phl and stably expressing dyn1(WT)-mCherry and shown on bottom.
(MOV)

Video S2 **TfR5 (left) and lifeact-mCherry (right) dynamics before, during, and after exposure to latrunculin B.** Dynamic actin foci, a portion of which co-localize with endocytic events, disappear upon the addition of 2.5 µM latrunculin B. A perfusion glitch occurs at 35 min. These frames were removed from the analysis.
(MOV)

Video S3 **TfR7 (left) and Dyn1(WT)-mCherry (right) dynamics before, during, and after exposure to latrunculin-B.**
(MOV)

Video S4 **TfR7 (left) Dyn1(T65A)-mCherry (right) dynamics before, during, and after exposure to latrunculin-B.**
(MOV)

Video S5 **TfR7 (left) Endo2-mCherry (right) dynamics before, during, and after exposure to latrunculin-B.**
(MOV)

Video S6 **TfR7 (left) GAK-mCherry (right) dynamics before, during, and after exposure to latrunculin-B.**
(MOV)

## Abbreviations

BIN1: BIN/Amphiphysin/RVS domain containing protein 1
CCS: clathrin-coated structures
CCV: clathrin-coated vesicle
CME: clathrin-mediated endocytosis
EM: electron microscopy
endo2: endophilin2
F-actin: filamentous actin
FRAP: fluorescence recovery after photobleaching
GAK: G-cyclin associated kinase
GTP: guanosine triophosphate
GTPase: guanosine triophosphate hydrolase
HA: hemagglutinin antigen
N-BAR: N-terminal containing BIN/Amphiphysin/RVS domain
TfR: transferrin receptor
TIR-FM: total internal reflection fluorescence microscopy
WT: wild type
