# Supplementary figures and images for: A Feedback Loop between Dynamin and Actin Recruitment during Clathrin-Mediated Endocytosis
Source: PLoS Biol. 2012 Apr 10;10(4):e1001302. doi: 10.1371/journal.pbio.1001302 (PMC3323523; doi:10.1371/journal.pbio.1001302)

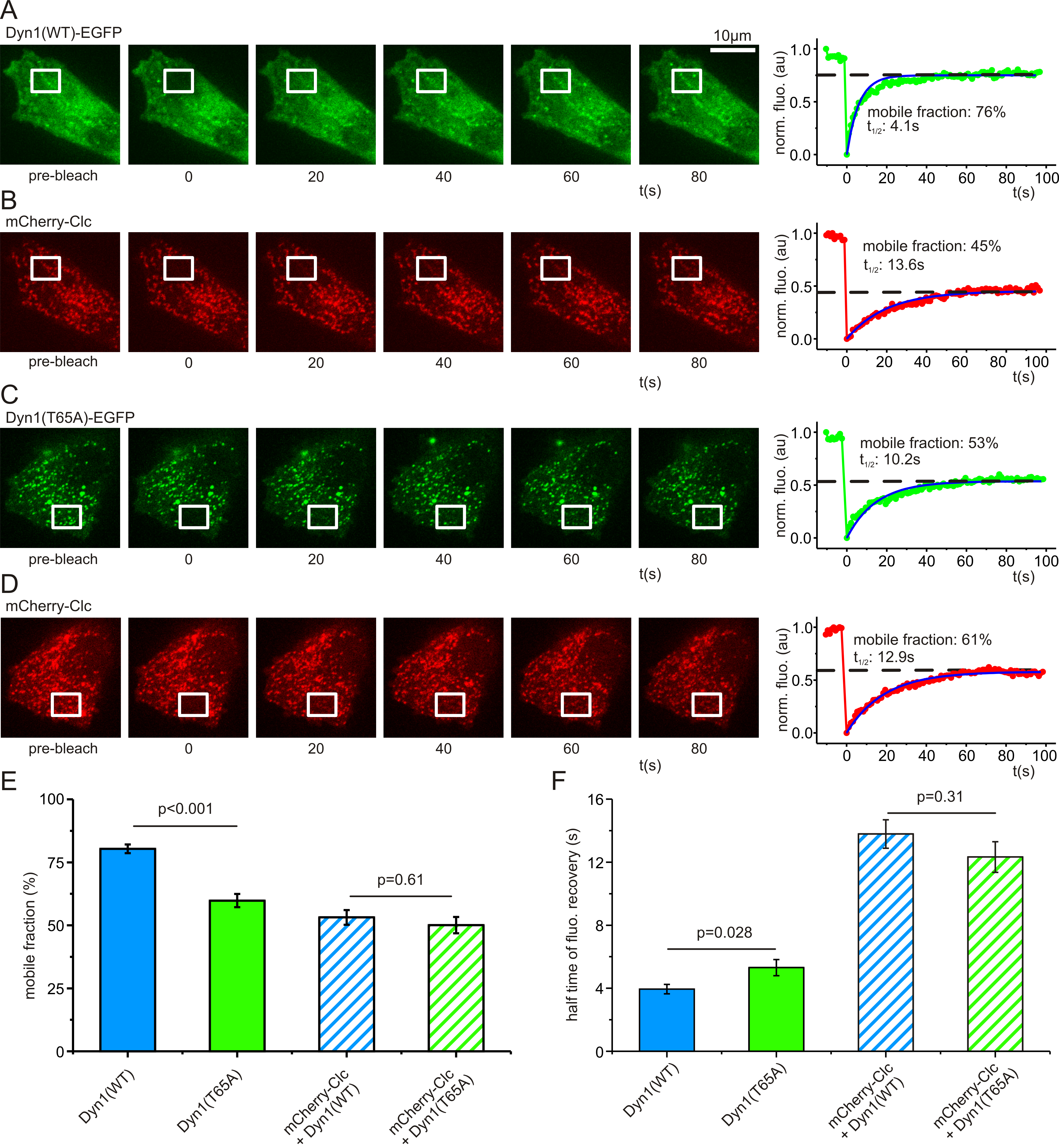

Supplement: Figure S1 — The turnover and mobility of Dyn1(WT/T65A)-eGFP and mCherry-Clc analyzed by FRAP. (A and B) An NIH-3T3 cell co-transfected with dyn1(WT)-EGFP (A) and mCherry-Clc (B). The mobility and turnover of dyn1 and Clc was analyzed using spinning disk confocal and FRAP. A 3-µm2 region (white box) containing CCSs was selected and bleached. The recovery of the fluorescent signal was analyzed to determine the mobile fraction and half time of recovery of dyn1(WT) and Clc (fluorescent time course in (A) and (B), see Methods for details of analysis). (C and D) NIH-3T3 cells co-transfected with dyn1(T65A)-EGFP (C) and mCherry-Clc (D) analyzed with FRAP (fluorescent time course in (C) and (D)). (E) The average mobile fraction, as determined by FRAP, of dyn1(WT) (n = 20 cells) and dyn1(T65A) (n = 17 cells). On average Dyn1(T65A) had a decreased mobile fraction in comparison to dyn1(WT) (59% versus 80% mobile fraction, respectively; Student's t test p<0.001) suggesting a significantly increased association with CCS. The mobility of mCherry-Clc was also analyzed and found not to be significantly different in cells expressing dyn1(WT) or dyn1(T65A) (53% versus 51% mobile fraction, respectively; Student's t test p = 0.61). (F) The average half time of fluorescence recovery for dyn1(WT) and dyn1(T65A). Dyn1(T65A) had a slower recovery time than dyn1(WT) (5.3 s versus 3.9 s, respectively; Student's t test p = 0.027). The recovery time of mCherry-Clc was not found to be significantly different in cells expressing dyn1(WT) or dyn1(T65A) (13.8 s versus 12.4 s, respectively; Student's t test p = 0.31). Error bars represent standard error of the mean (SEM). (TIF) [file pbio.1001302.s001.tif]

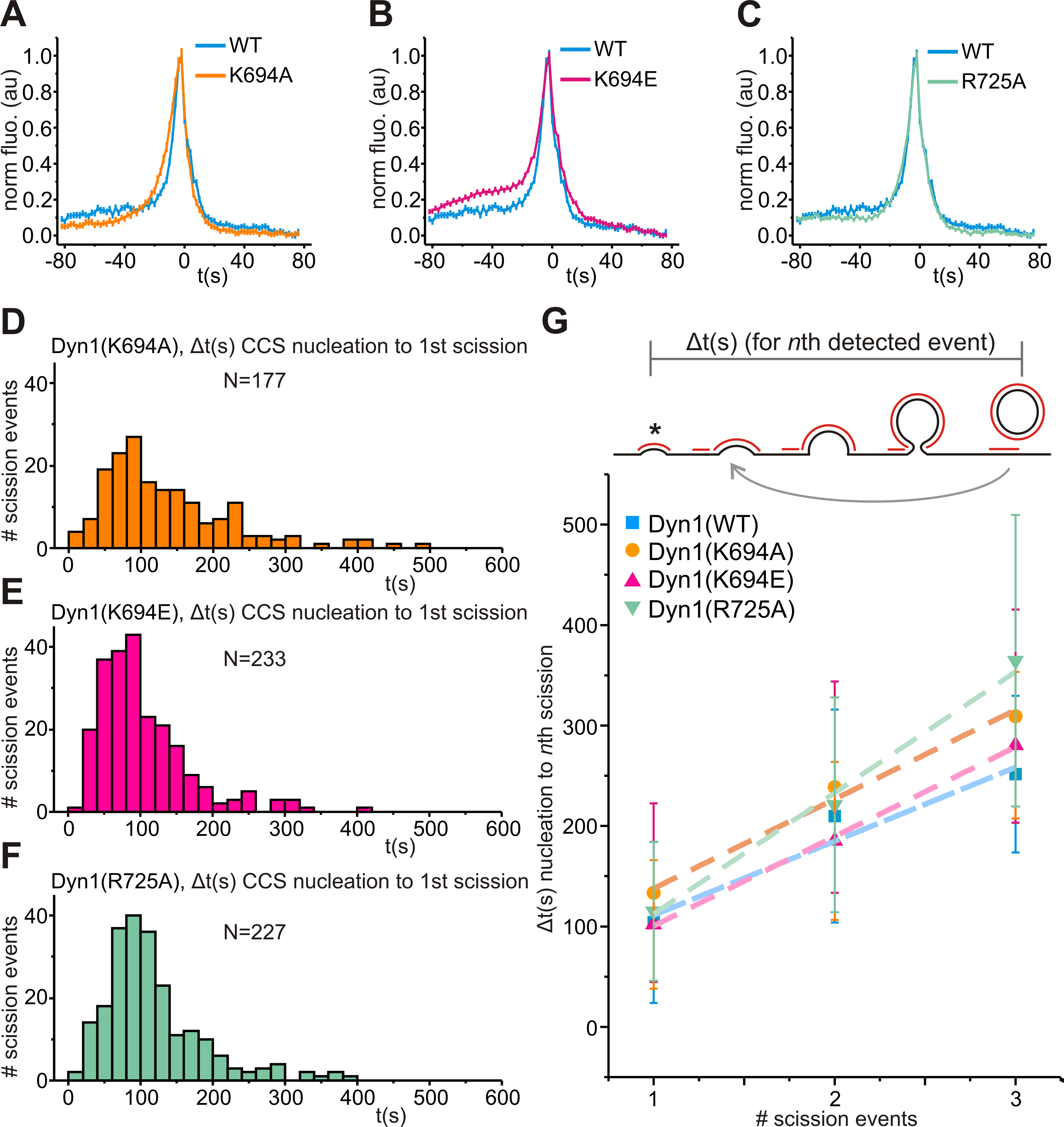

Supplement: Figure S2 — The effects of dynamin GED domain mutants on dynamin recruitment kinetics and de novo CCS lifetime. (A–C) Comparison of dyn1(WT)-mCherry and GED domain mutant recruitment signatures. The recruitment signatures of dyn1(K694A)-mCherry (A), dyn1(K694E)-mCherry (B), and dyn1(R725A)-mCherry (C) displayed similar recruitment kinetics to scission as dyn1(WT)-mCherry. (D–F) Histograms of the time difference from de novo CCS nucleation to first detected scission events in cells expressing dyn1(K694A)-mCherry (D), dyn1(K694E)-mCherry (E), and dyn1(R725A)-mCherry (F). (G) Point mutations in the GED region of dynamin had little effect upon the average time from CCS nucleation to the nth scission event (see text for explanation of measurement). Expression of dyn1(K694A/K694E/R725A)-mCherry did not significantly increase the time from nucleation to the nth scission event compared to NIH-3T3 cells expressing dyn1(WT)-mCherry. Error bars represent standard deviation. (TIF) [file pbio.1001302.s002.tif]

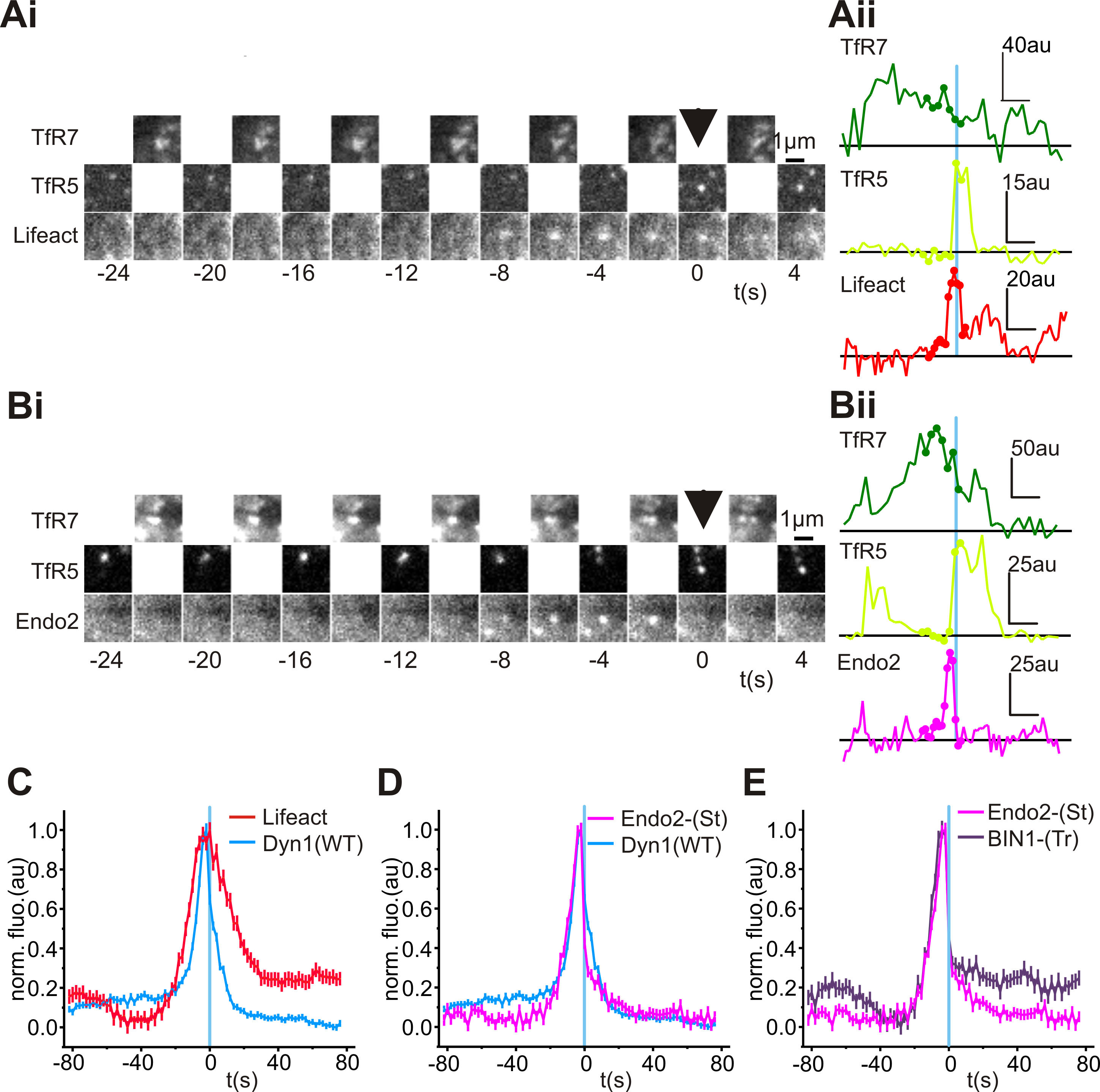

Supplement: Figure S3 — Actin and N-BAR proteins were recruited at the same time as Dynamin to sites of scission but with distinct kinetics. (A) Actin was recruited in the moments leading up to vesicle appearance. (Ai) A time series of an example scission event in a cell expressing lifeact-mCherry showing the recruitment of F-actin to sites of scission. (Aii) Fluorescent measurements (dark green, TfR7; light green, TfR5; red, lifeact) of the event shown in (Ai). Dots correspond to the images shown in (Ai). (B) The N-BAR protein endo2 was recruited in the seconds preceding vesicle appearance. (Bi) Time series of an example scission event in an NIH-3T3 cell expressing endo2-mCherry. (Bii) Fluorescence measurements from the time series shown in (Ai) (dark green, TfR7; light green, TfR5; purple, endo2). Dots correspond to the time series shown. Horizontal scale bars in (A) and (B) corresponds to 20 s, and blue line represents t = 0, the moment of scission. (C) Ensemble recruitment signature of lifeact-mCherry (6 cells, 788 events) compared to dyn1(WT) (11 cells 3157 events). Lifeact recruitment was measured in NIH-3T3 cells transiently expressing lifeact-mCherry (D). Comparison of endo2-mCherry and dyn1(WT)-mCherry recruitment signatures. Endo2-mCherry recruitment was measured in NIH-3T3 cells stably (St) expressing endo2-mCherry (6 cells, 2608 events). (E) N-BAR proteins BIN1 and endo2 showed similar recruitment kinetics to scission. The normalized recruitment signature from NIH-3T3 cells stably expressing endo2-mCherry and transiently (Tr) expressing BIN1-mCherry (11 cells, 3,024). (TIF) [file pbio.1001302.s003.tif]

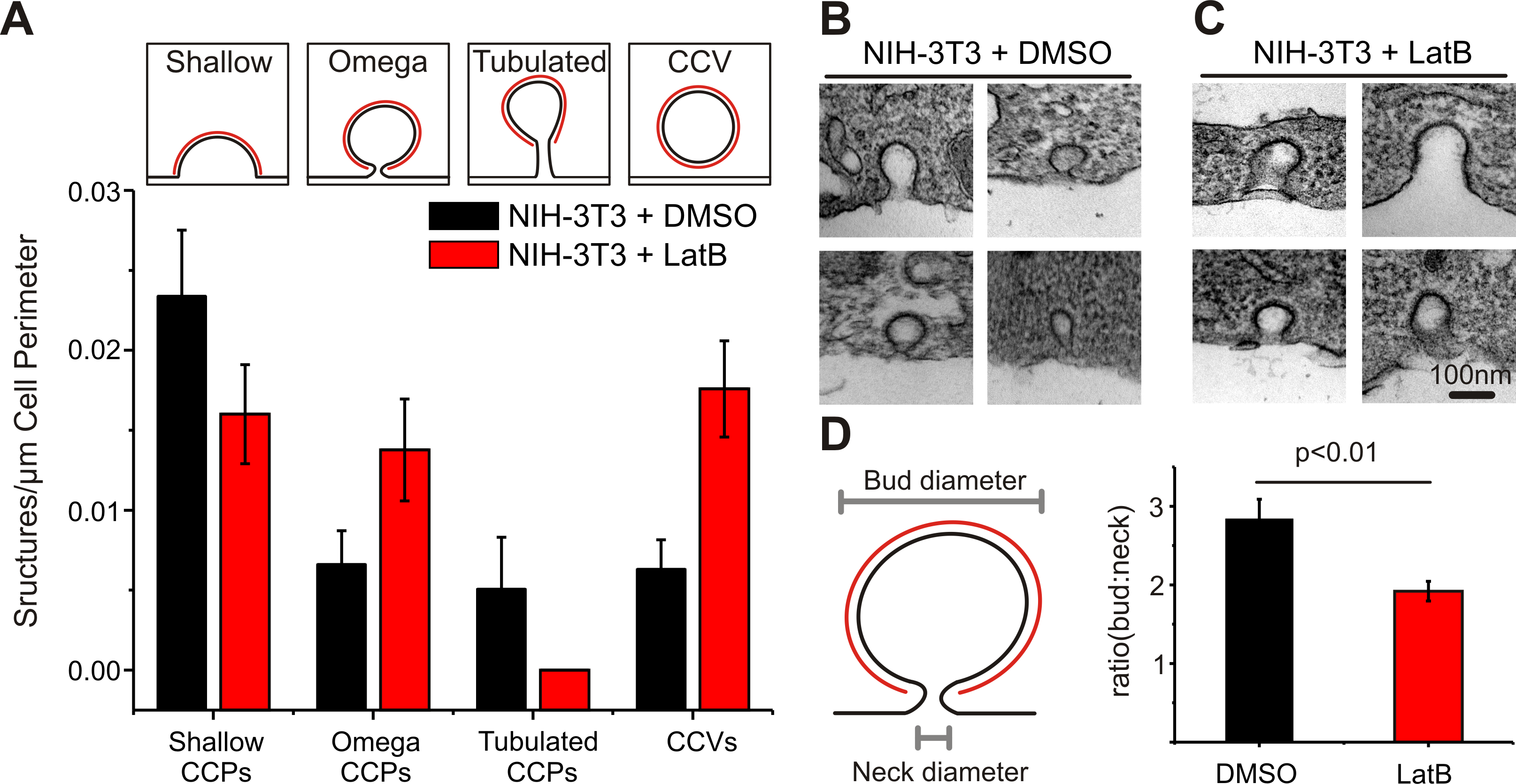

Supplement: Figure S4 — Ultrastructure of CCS in the presence of latrunculin B. (A) Morphometric analysis of coated pit profiles in NIH-3T3 exposed to DMSO or latrunculin B. Insets show sketches of the coated pit profiles included in each category. Data were obtained from >30 randomly selected cell profiles. Error bars represent standard error of the mean. (B and C) Example images of omega-shaped coat pit profile from NIH-3T3 exposed to DMSO or latrunculin B. Omega-shaped coated pits in cell exposed to latrunculin B appear to have wider necks. (D) Quantification of the ratio between the bud and neck diameter of omega-coated pits suggest an inhibition of neck constriction in the presence of latrunculin B. Measurement made from a subset of >30 structures classified as omega-coated pits. Error bars represent standard error of the mean. Student's t test, p = 0.0097. (TIF) [file pbio.1001302.s004.tif]

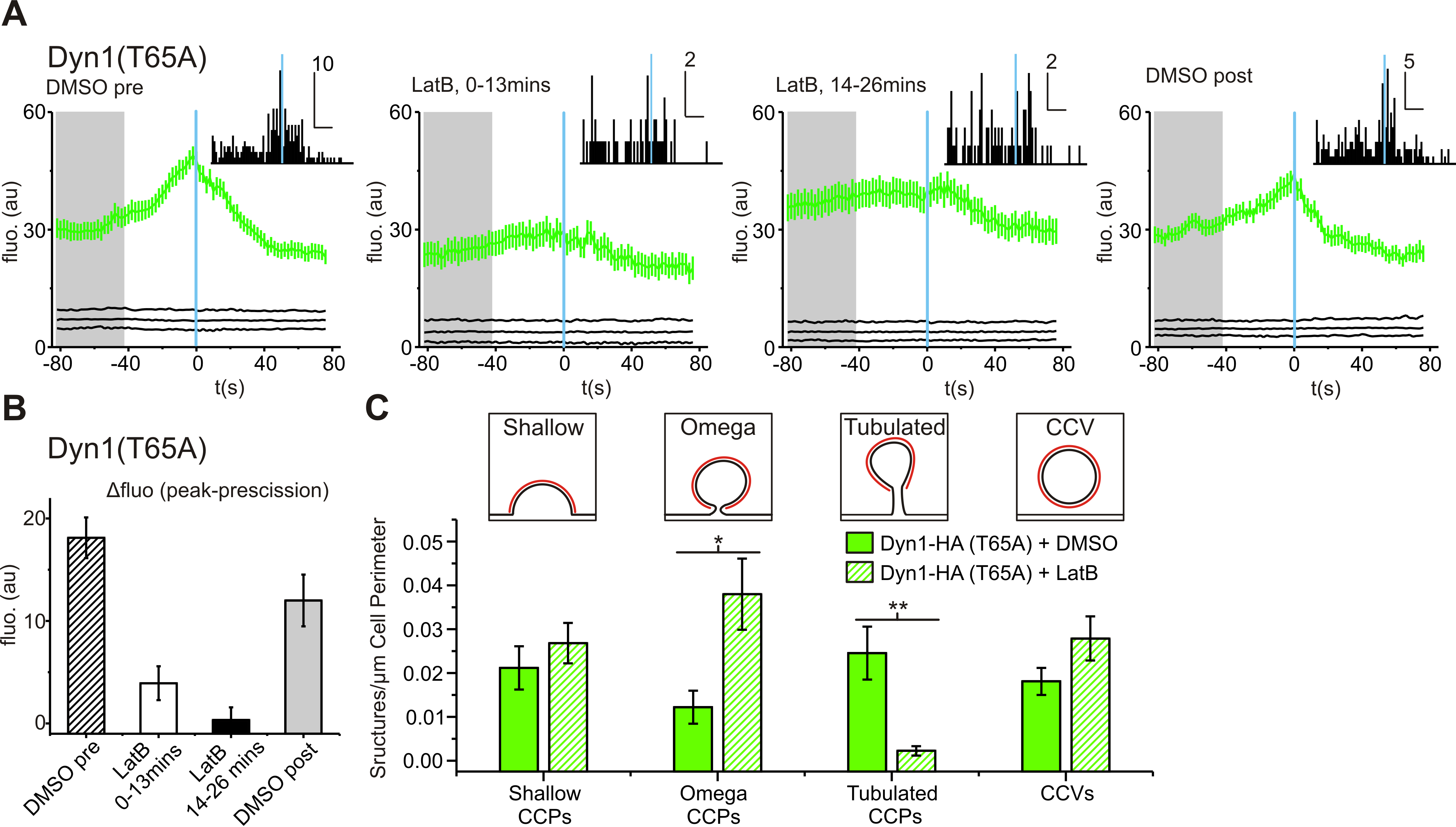

Supplement: Figure S5 — Actin remodeling is not required for the concentration of dyn(T65A)-mCherry at CCSs. (A) Acute inhibition of actin decreased the amplitude of peak dyn1(T65A)-mCherry recruitment at scission, but does not perturb the pre-scission recruitment. Inset: histograms of peak dyn1(T65A)-mCherry fluorescence for each scission event that composed the ensemble average (horizontal scale bar indicate the number of event, vertical scale bar correspond to 20 s). Recruitment traces from five cells: (DMSO pre-exposure: 383 events, 0–13 min exposure to latrunculin-B; 116 events, 14–26 min exposure; 178 events, and DMSO washout: 342 events). (B) Latrunculin-B exposure decreased the peak amplitude of dyn1(T65A), in a similar manner to dyn1(WT) (see Figure 6). See Materials and Methods for details of quantification (C). Morphometric analysis of coated pit profiles in NIH-3T3 expressing dyn1(T65A)-HA exposed to DMSO or latrunculin B. As with untransfected NIH-3T3 cells (see Figure S4), treatment with latrunculin-B resulted in an increase in the frequency of “omega”-shaped coated pit profiles. Insets show sketches of the coated pit profiles included in each category. Data were obtained from >30 randomly selected cell profiles. Error bars represent standard error of the mean. (TIF) [file pbio.1001302.s005.tif]

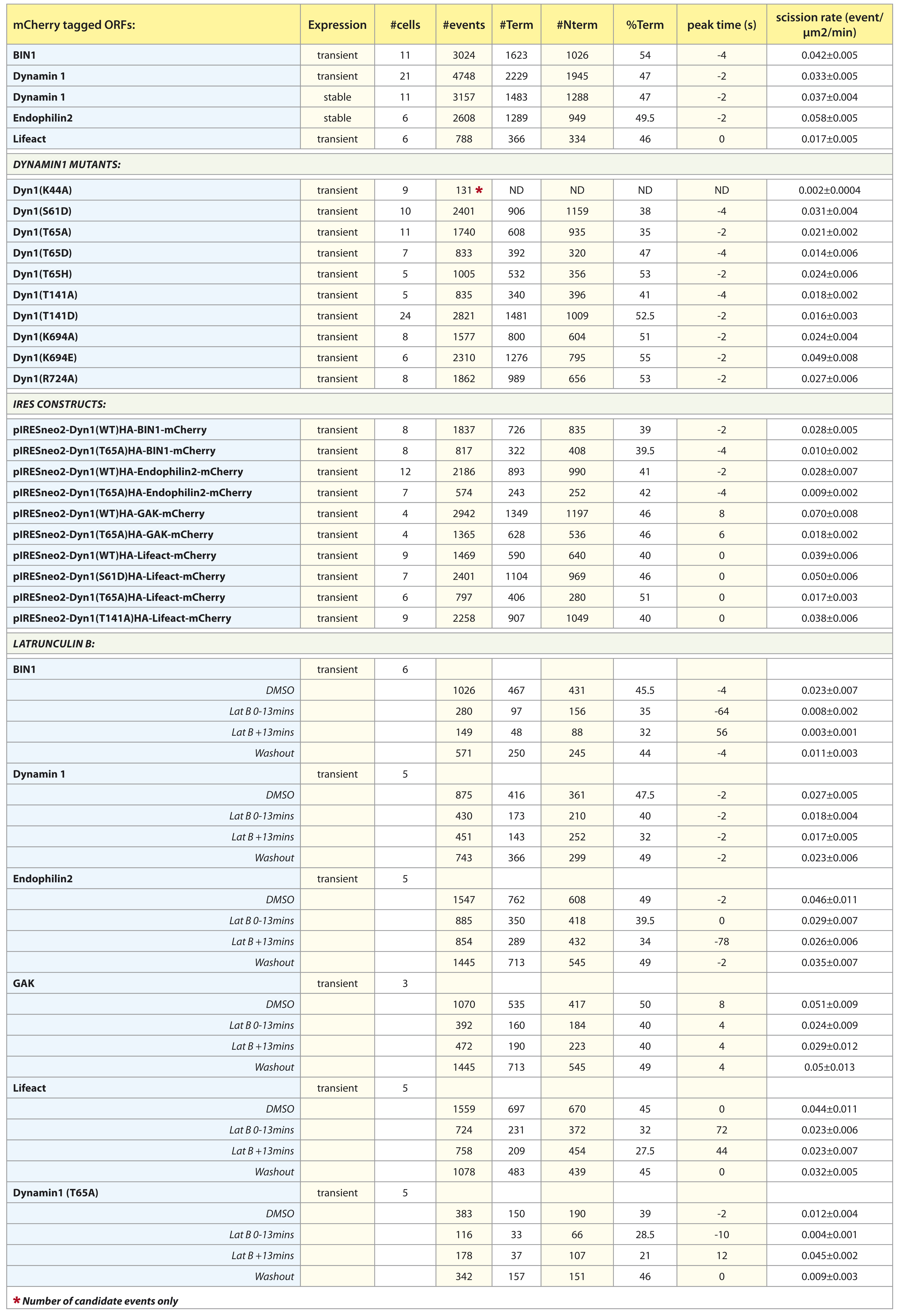

Supplement: Table S1 — Parameters for the cells used in this study. Expression: whether the construct was transiently or stably expressed. #cells, number of cells recorded; #events, total number of events detected and analyzed; #Term, number of events classified as terminal, i.e., with disappearance of the CCS (see Methods); #Nterm, number of events classified as non-terminal (no CCS disappearance); %Term, percentage of terminal events; Peak time, time of maximum average fluorescence relative to CCV detection, in seconds; Scission rate, average over individual cells of the rate of event detection per µm2 per minute, error represents standard error of the mean. (JPG) [file pbio.1001302.s006.jpg]
